# Supplementary figures and images for: Glycolytic flux-signaling controls mouse embryo mesoderm development
Source: eLife. 2022 Dec 5;11:e83299. doi: 10.7554/eLife.83299 (PMC9771359; doi:10.7554/eLife.83299)

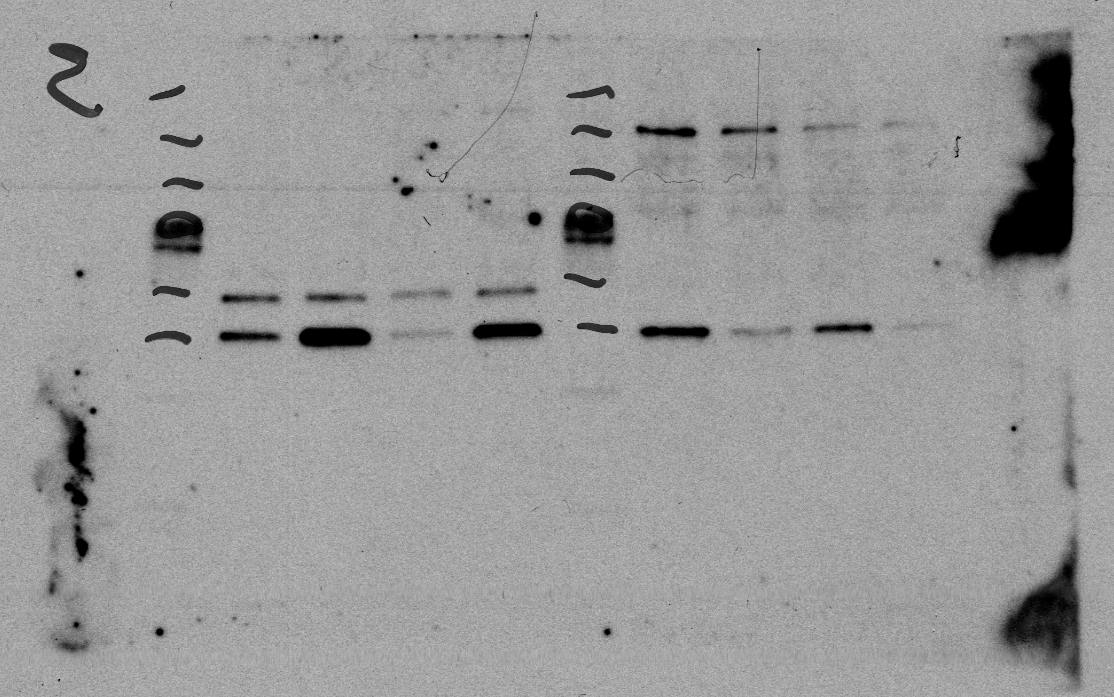

Supplement: Figure 6—source data 1. [file elife-83299-fig6-data1.zip › Aldoa.tiff]

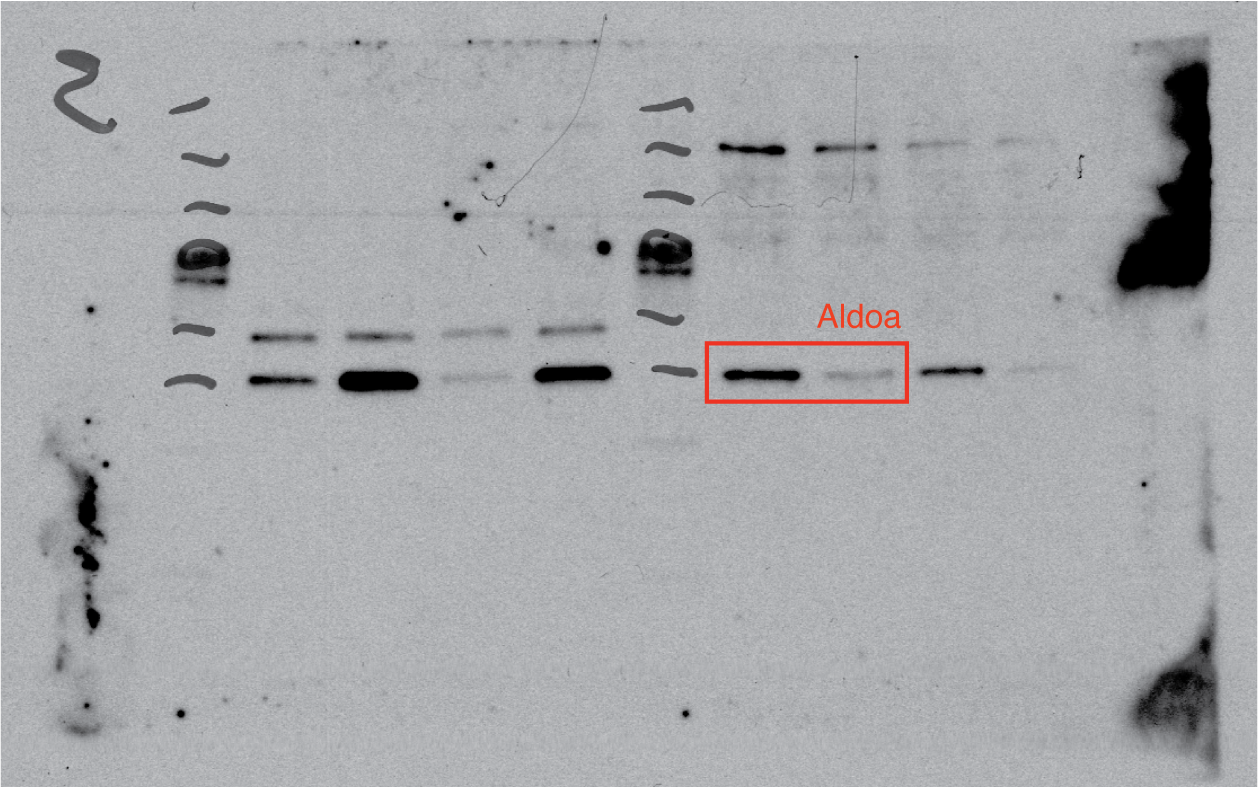

Supplement: Figure 6—source data 1. [file elife-83299-fig6-data1.zip › Aldoa_highlighted.tif]

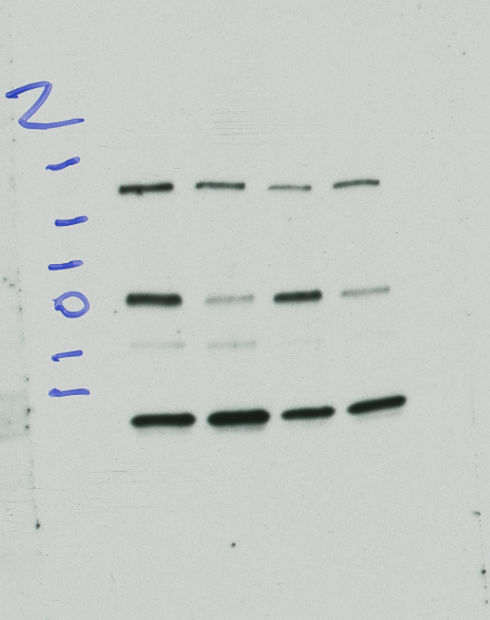

Supplement: Figure 6—source data 1. [file elife-83299-fig6-data1.zip › Gapdh.tiff]

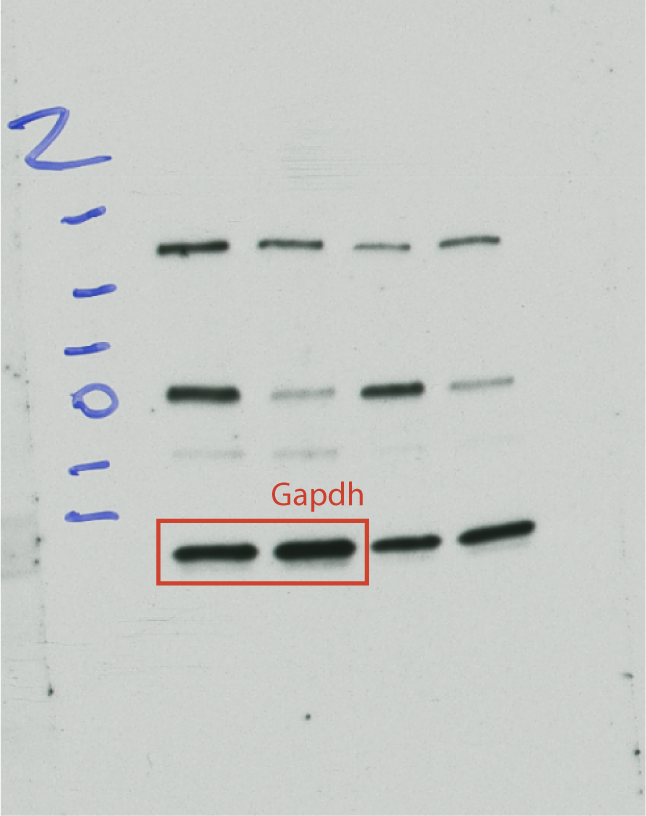

Supplement: Figure 6—source data 1. [file elife-83299-fig6-data1.zip › Gapdh_highlighted.tif]

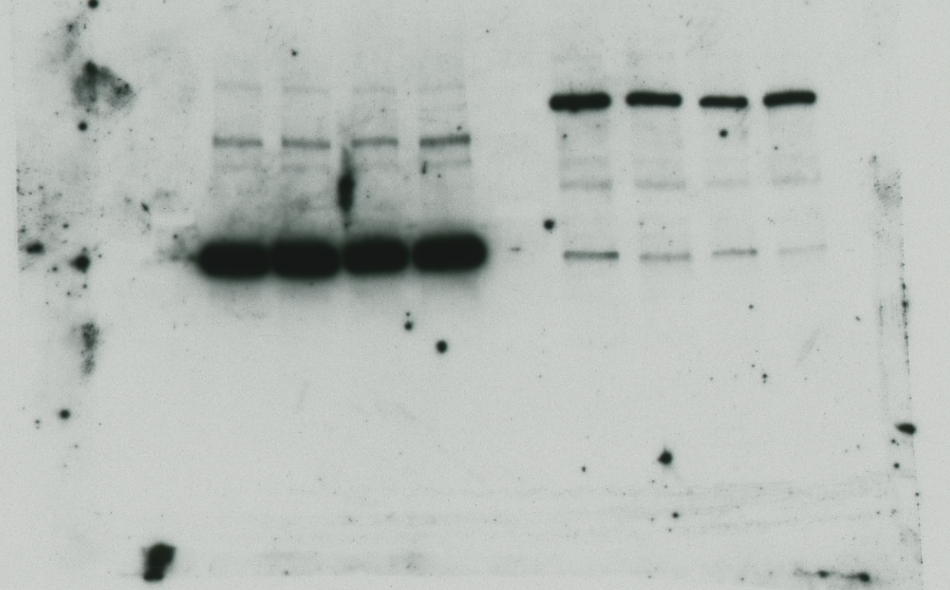

Supplement: Figure 6—source data 1. [file elife-83299-fig6-data1.zip › PKM.tiff]

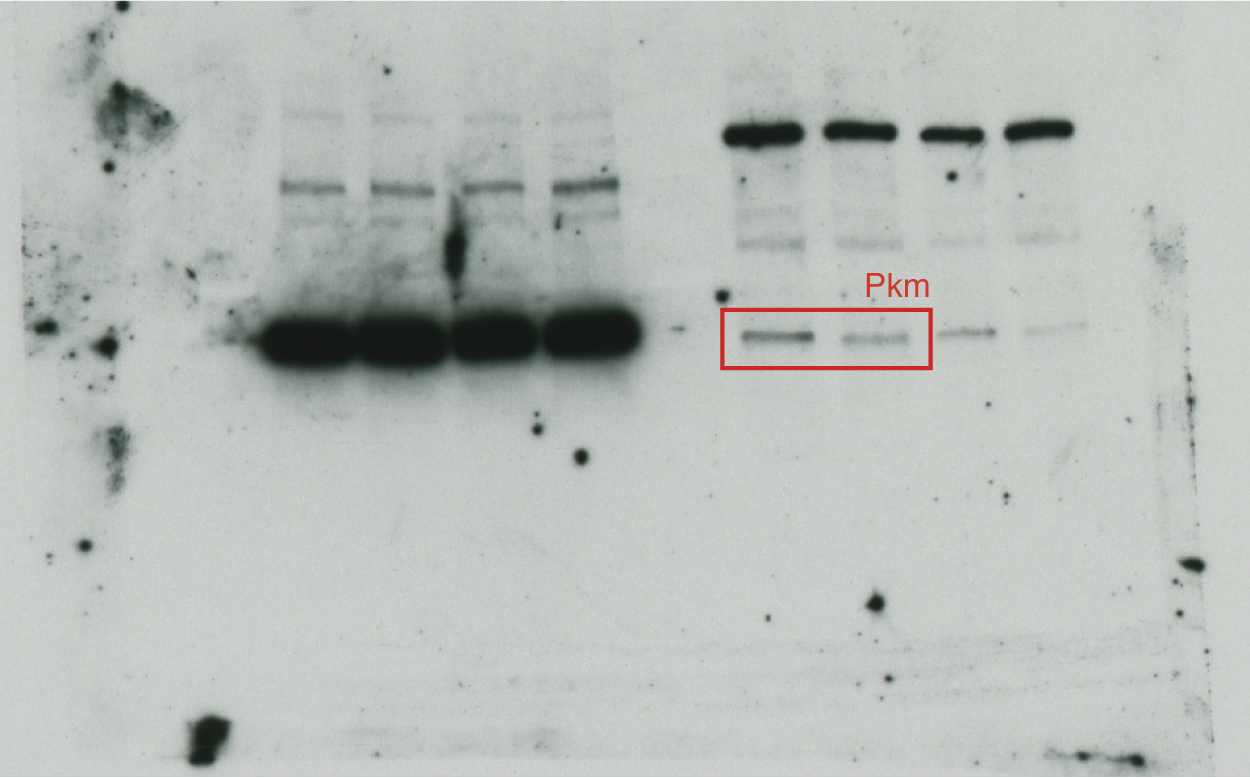

Supplement: Figure 6—source data 1. [file elife-83299-fig6-data1.zip › PKM_highlighted.tif]

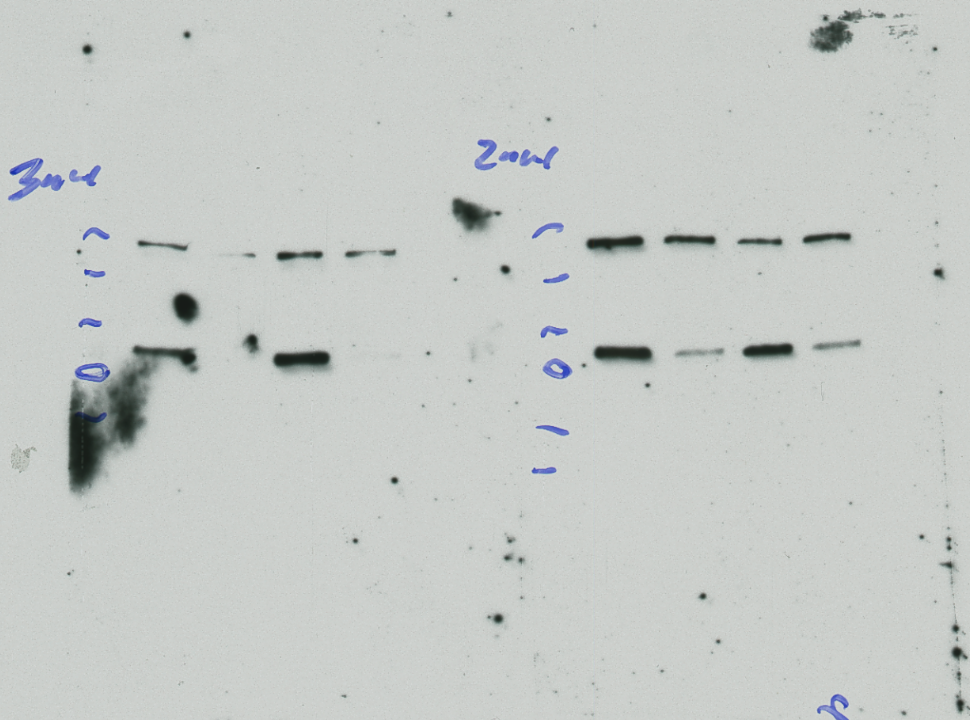

Supplement: Figure 6—source data 1. [file elife-83299-fig6-data1.zip › Top2b_Pfkl.tiff]

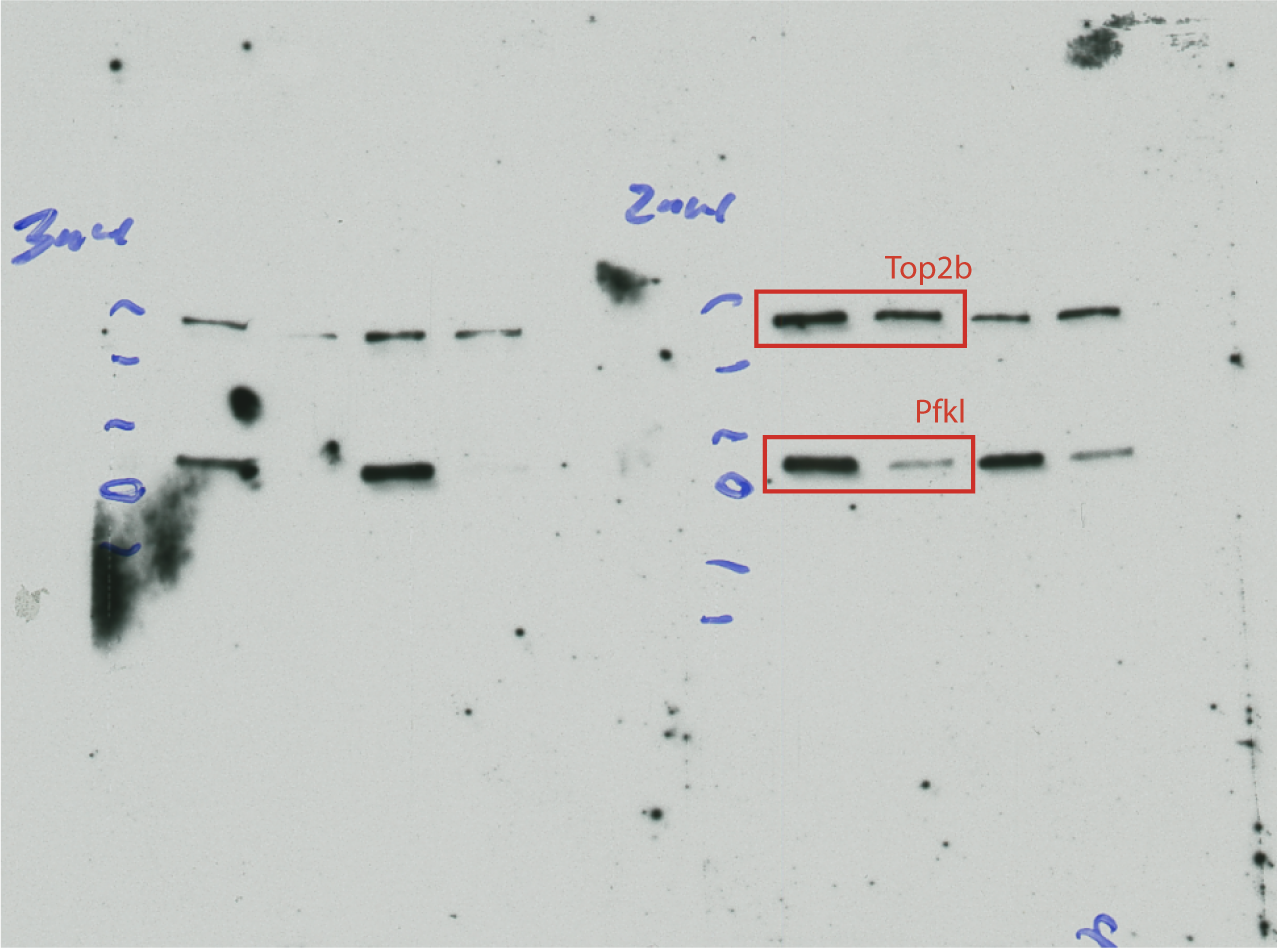

Supplement: Figure 6—source data 1. [file elife-83299-fig6-data1.zip › Top2b_Pfkl_highlighted.tif]

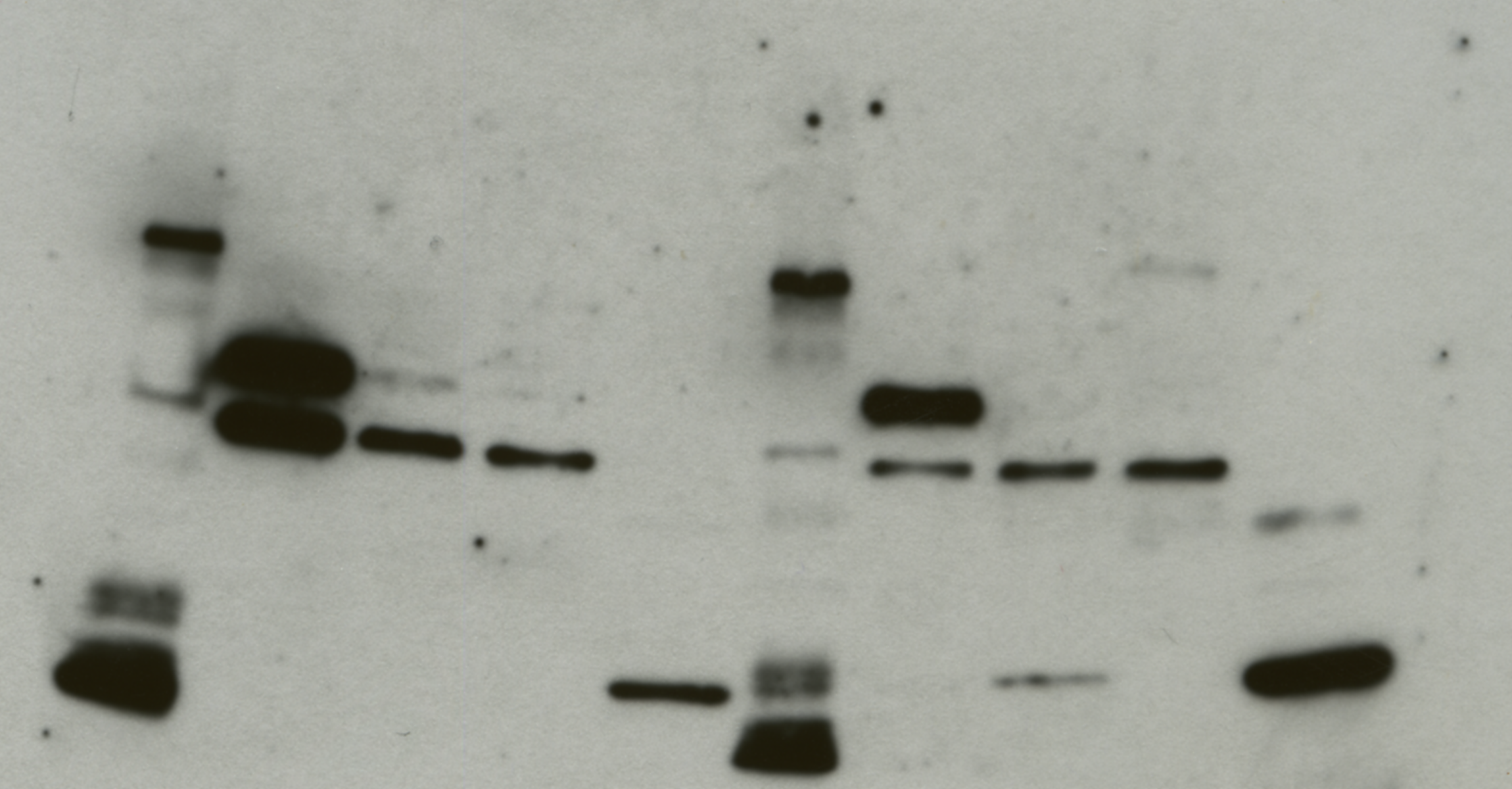

Supplement: Figure 6—figure supplement 1—source data 1. [file elife-83299-fig6-figsupp1-data1.zip › Aldoa_b-Tubulin_Histone-H2B.tiff]

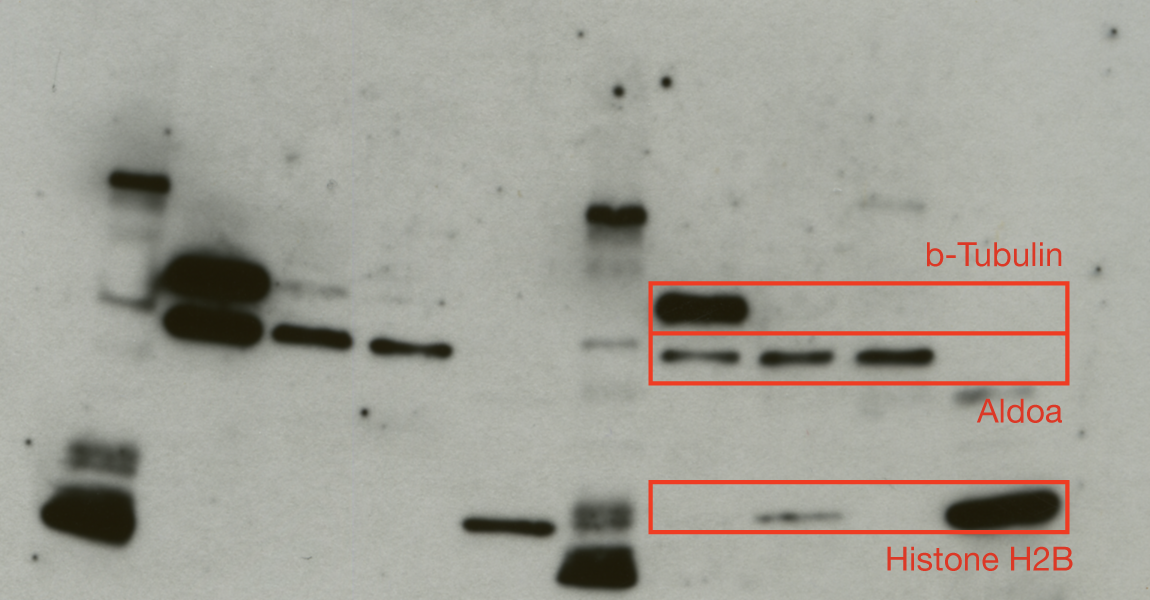

Supplement: Figure 6—figure supplement 1—source data 1. [file elife-83299-fig6-figsupp1-data1.zip › Aldoa_b-Tubulin_Histone-H2B_highlighted.tif]

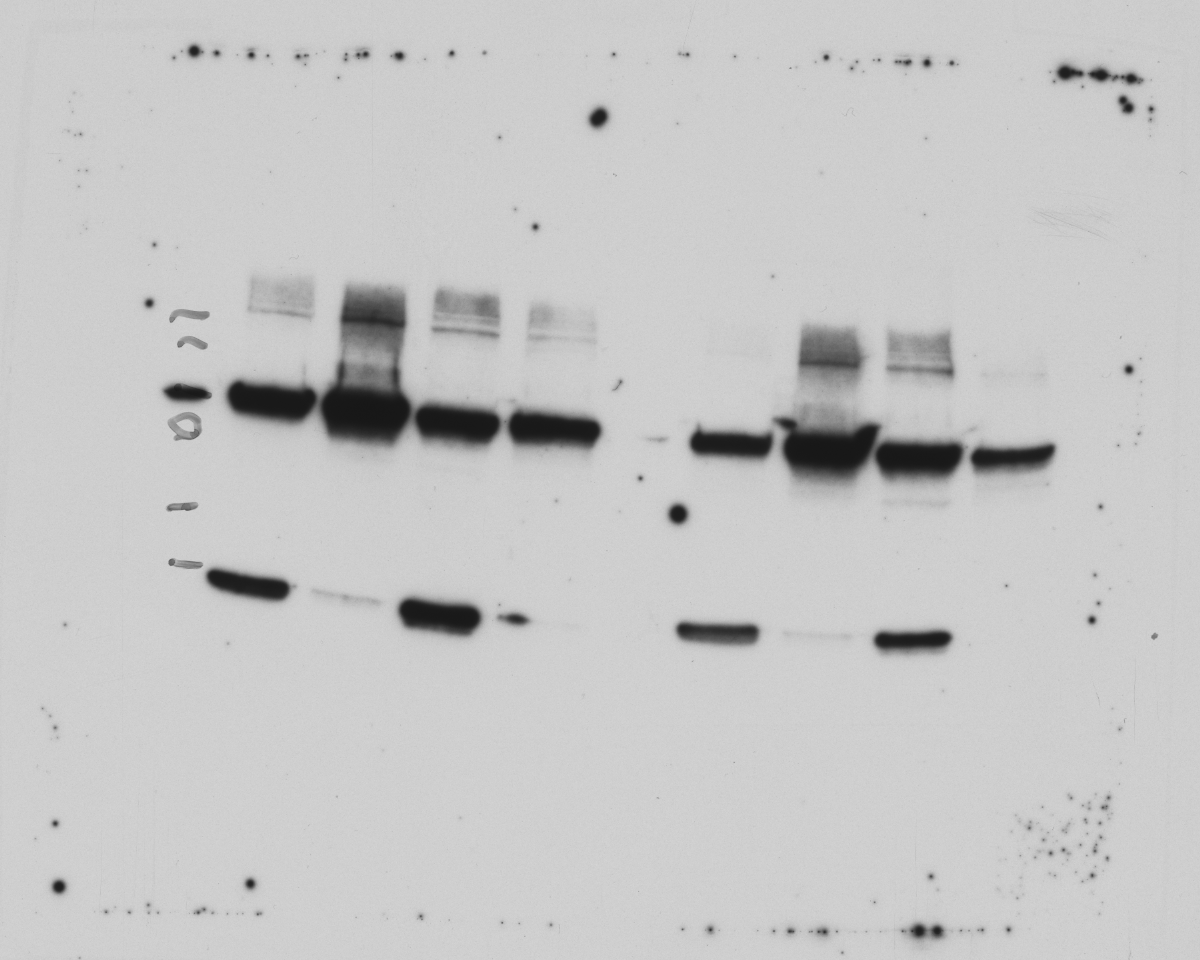

Supplement: Figure 6—figure supplement 1—source data 1. [file elife-83299-fig6-figsupp1-data1.zip › Gapdh.tiff]

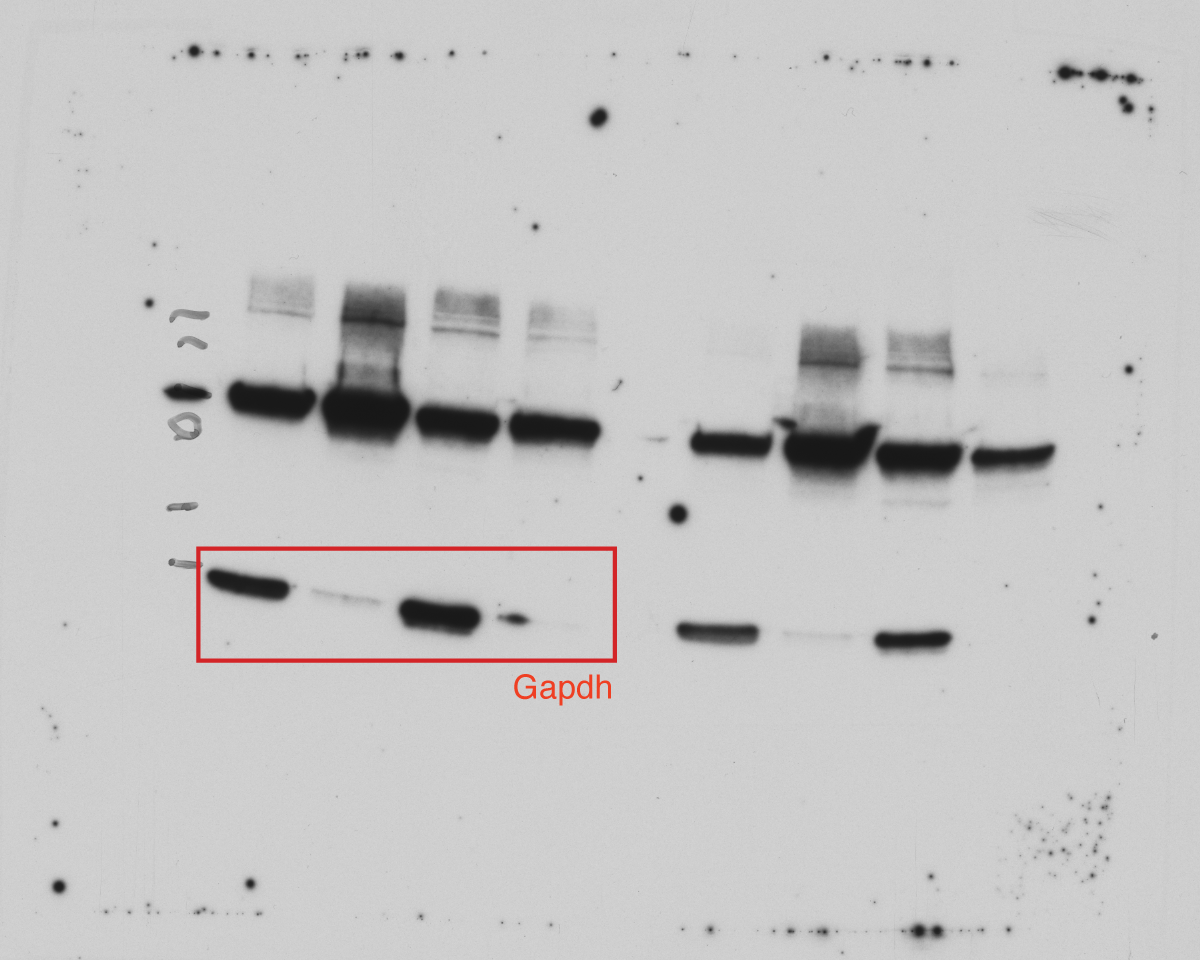

Supplement: Figure 6—figure supplement 1—source data 1. [file elife-83299-fig6-figsupp1-data1.zip › Gapdh_highlighted.tif]

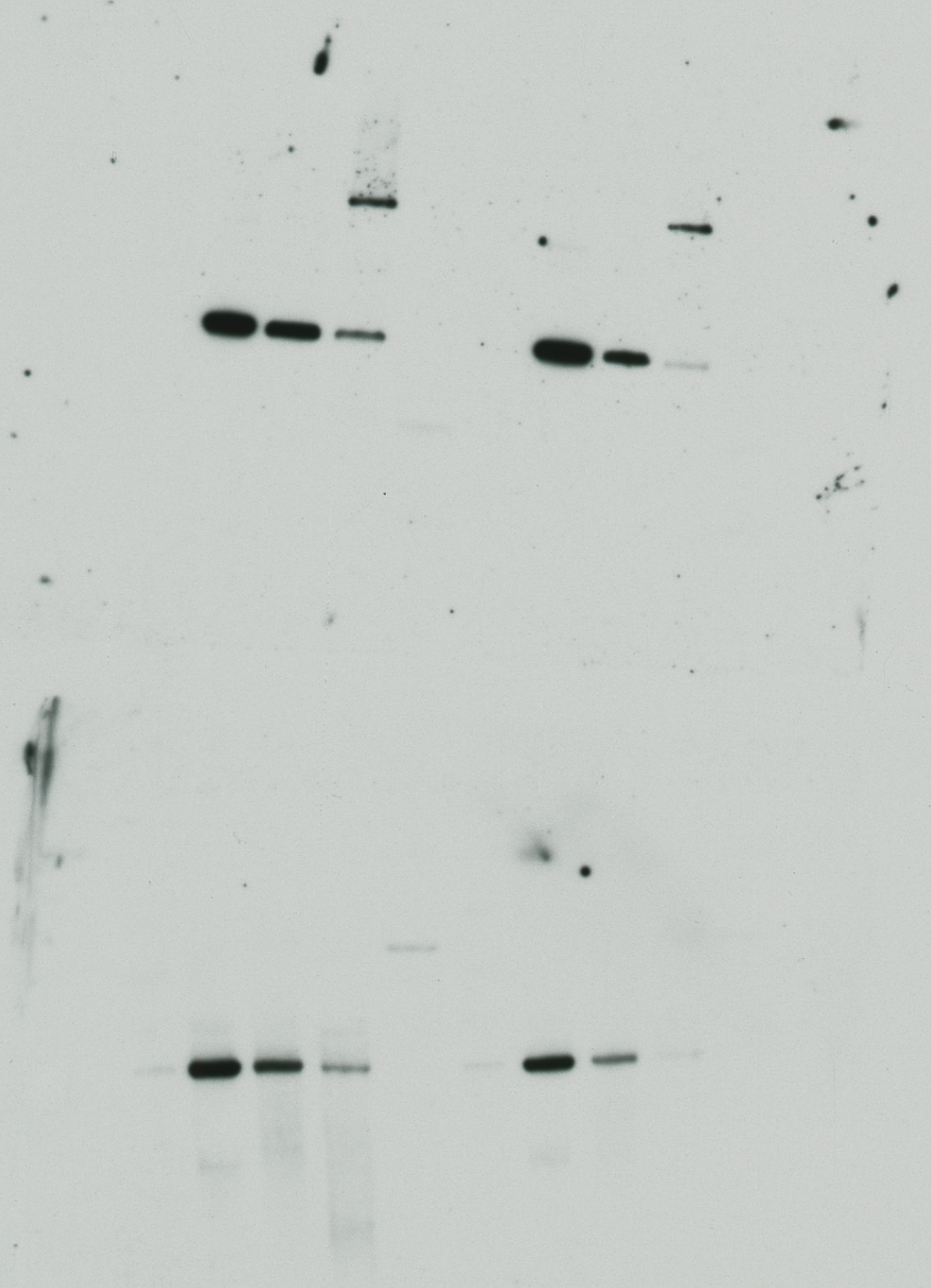

Supplement: Figure 6—figure supplement 1—source data 1. [file elife-83299-fig6-figsupp1-data1.zip › Pkm_Pfkl_Top2b.tiff]

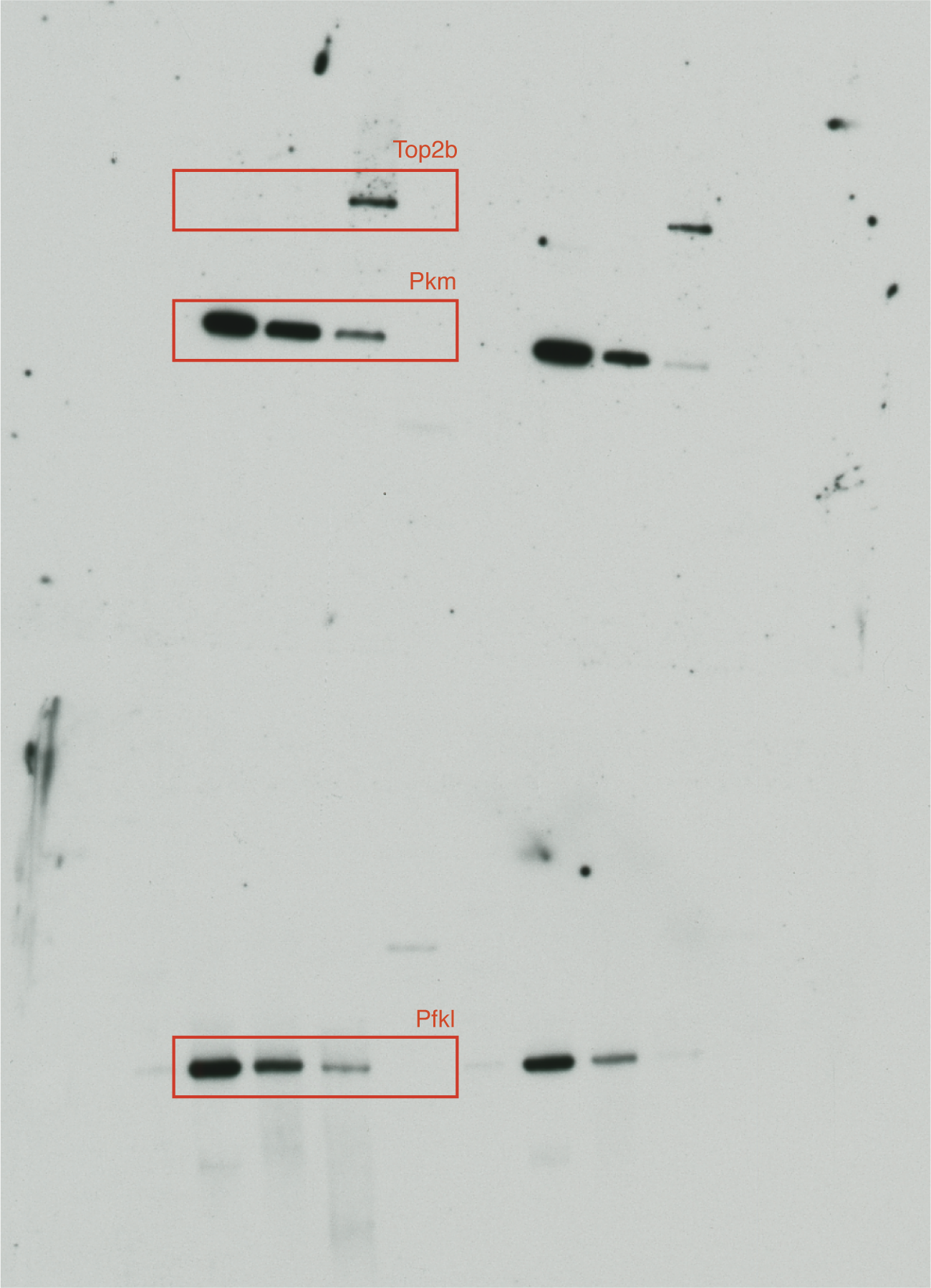

Supplement: Figure 6—figure supplement 1—source data 1. [file elife-83299-fig6-figsupp1-data1.zip › Pkm_Pfkl_Top2b_highlighted.tif]

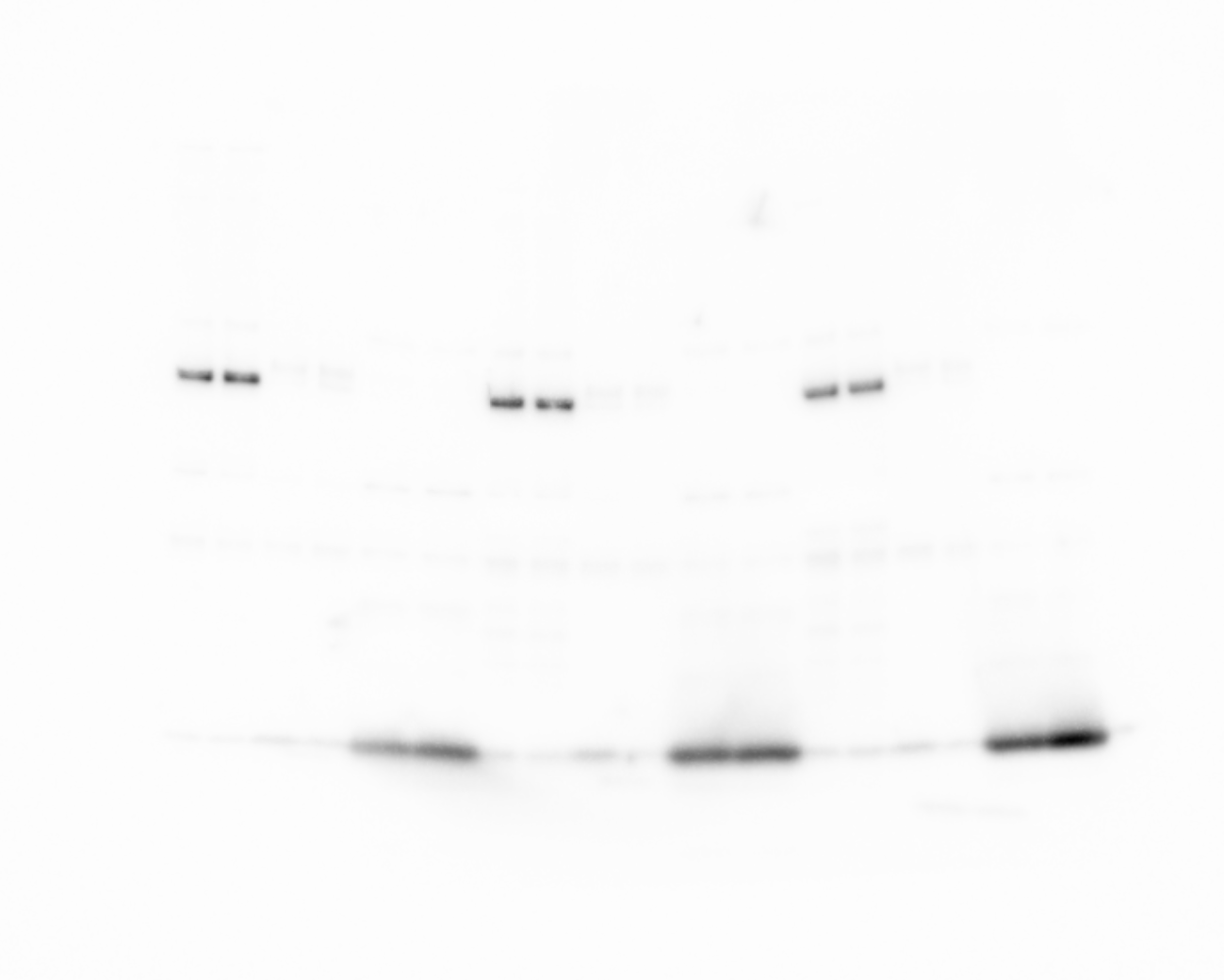

Supplement: Figure 7—source data 1. [file elife-83299-fig7-data1.zip › Hsp90_10mM-glucose.tif]

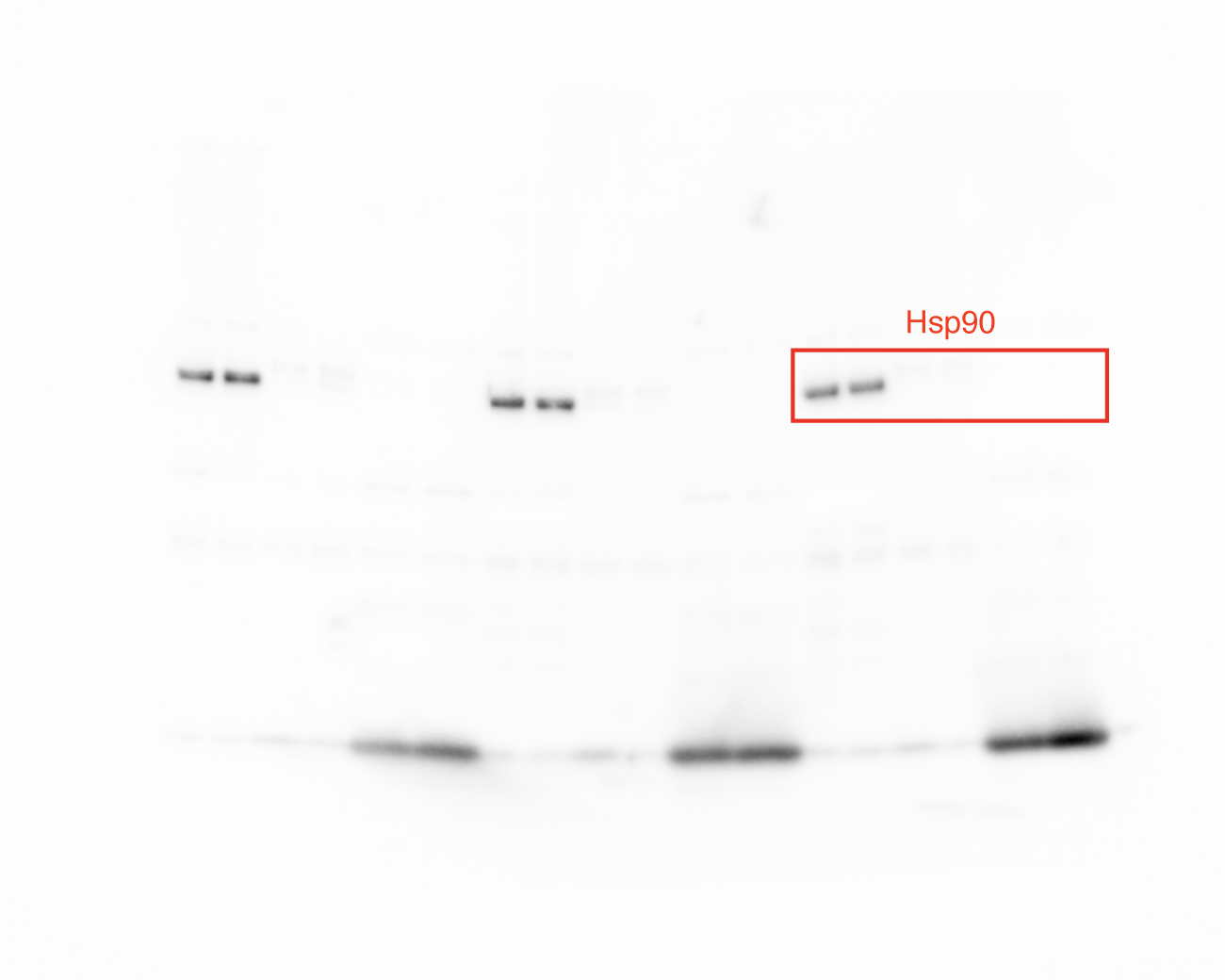

Supplement: Figure 7—source data 1. [file elife-83299-fig7-data1.zip › Hsp90_10mM-glucose_highlighted.tif]

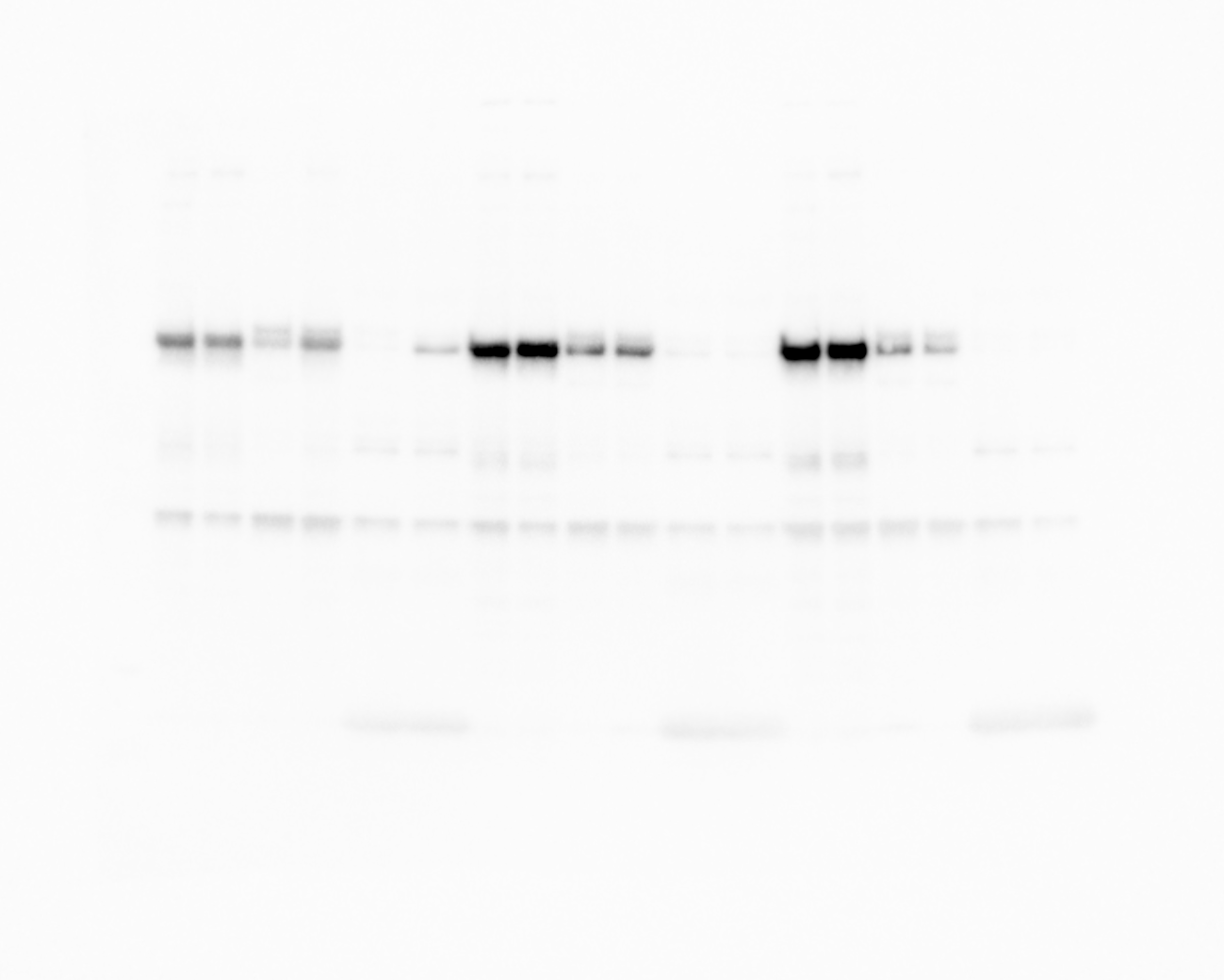

Supplement: Figure 7—source data 1. [file elife-83299-fig7-data1.zip › Hsp90_2mM-glucose.tif]

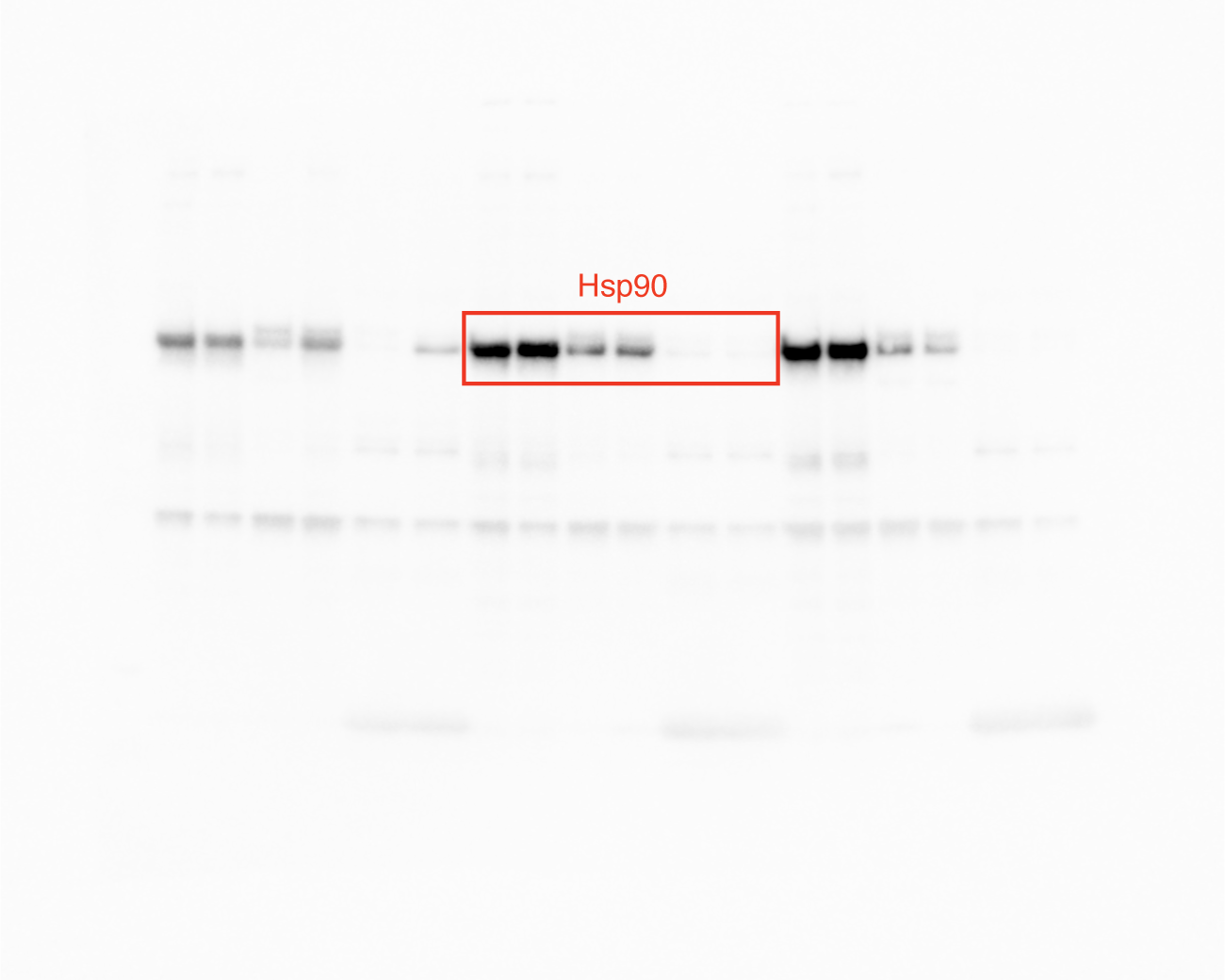

Supplement: Figure 7—source data 1. [file elife-83299-fig7-data1.zip › Hsp90_2mM-glucose_highlighted.tif]

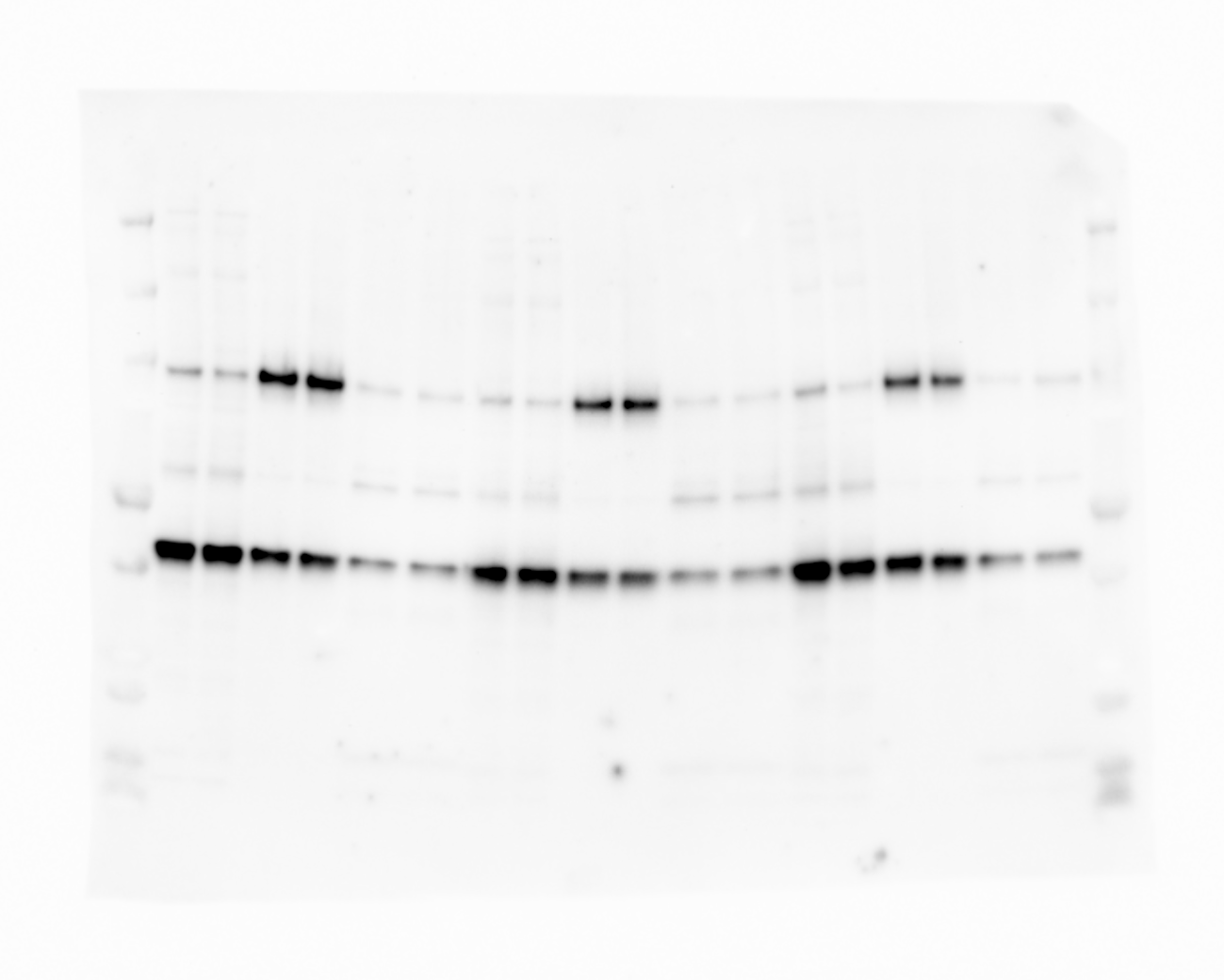

Supplement: Figure 7—source data 1. [file elife-83299-fig7-data1.zip › Na-K-ATPase_10mM-glucose.tif]

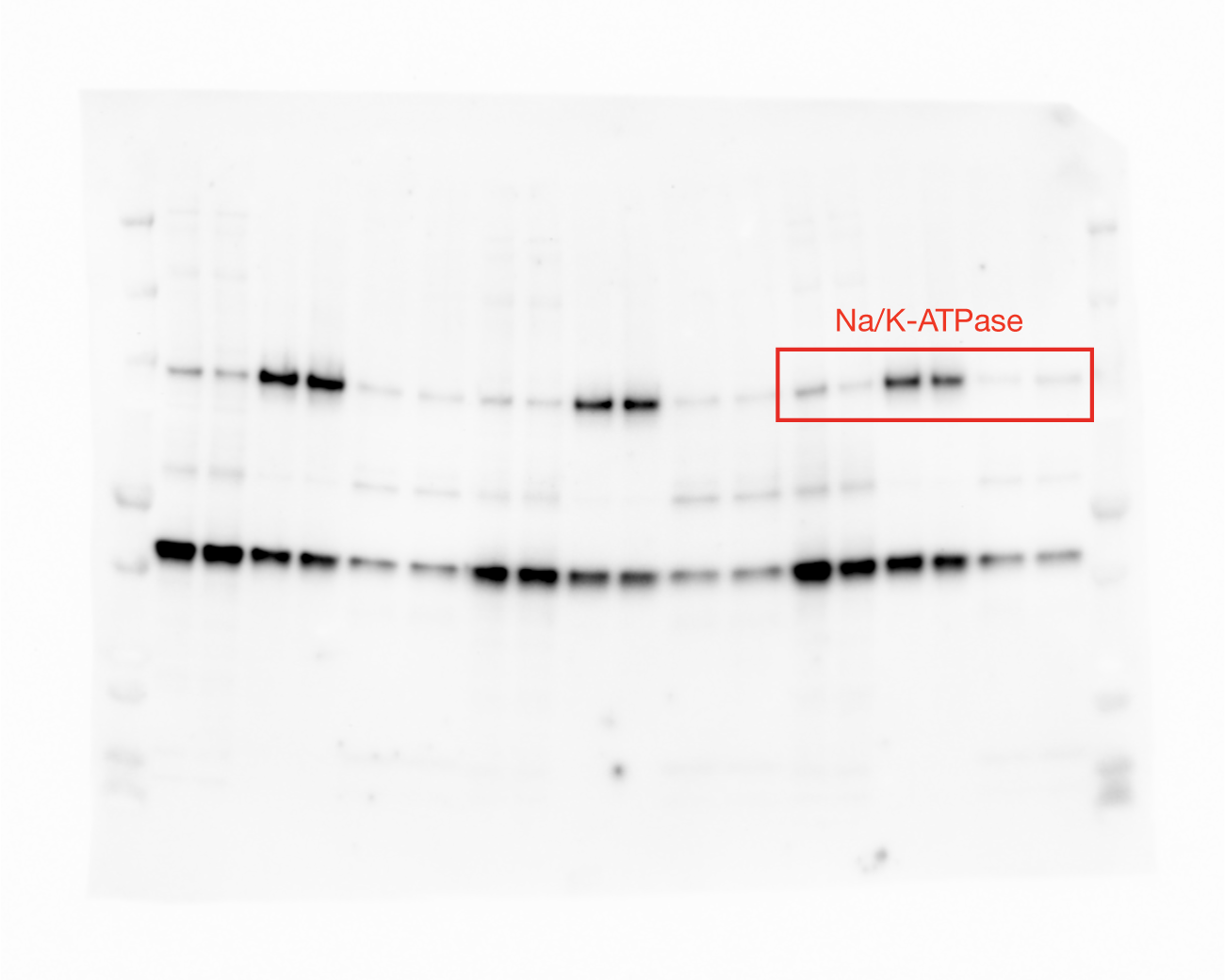

Supplement: Figure 7—source data 1. [file elife-83299-fig7-data1.zip › Na-K-ATPase_10mM-glucose_highlighted.tif]

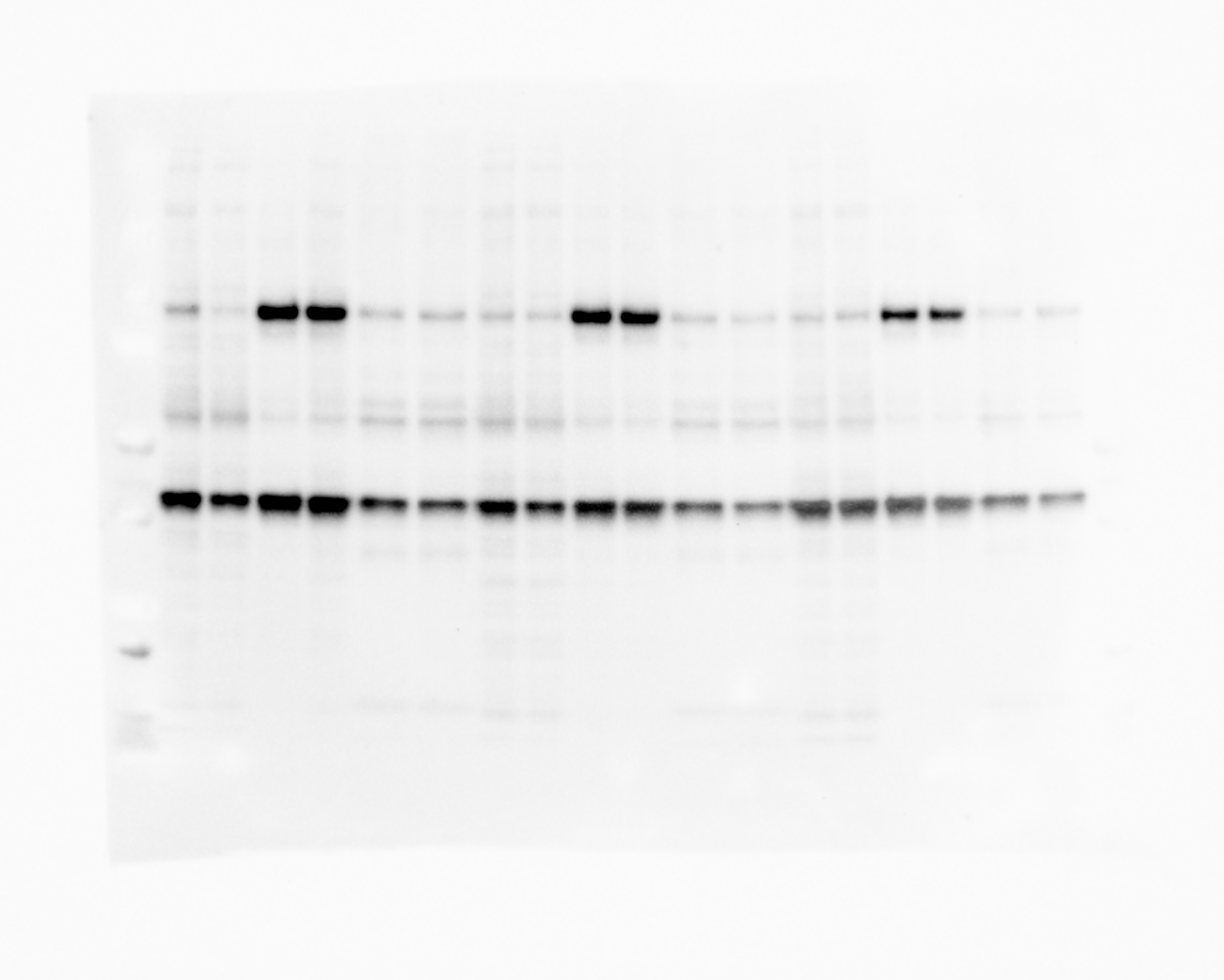

Supplement: Figure 7—source data 1. [file elife-83299-fig7-data1.zip › Na-K-ATPase_2mM-glucose.tif]

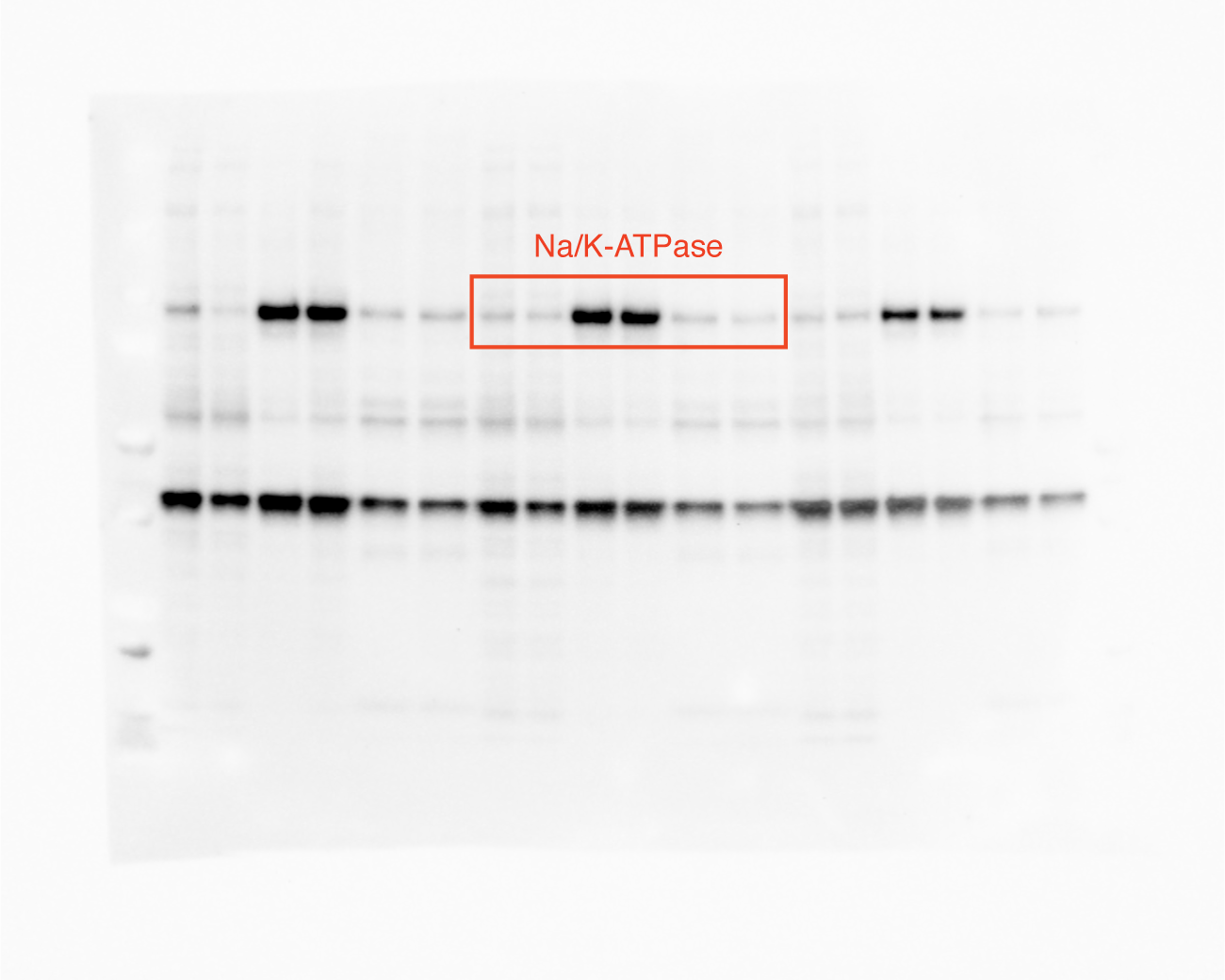

Supplement: Figure 7—source data 1. [file elife-83299-fig7-data1.zip › Na-K-ATPase_2mM-glucose_highlighted.tif]

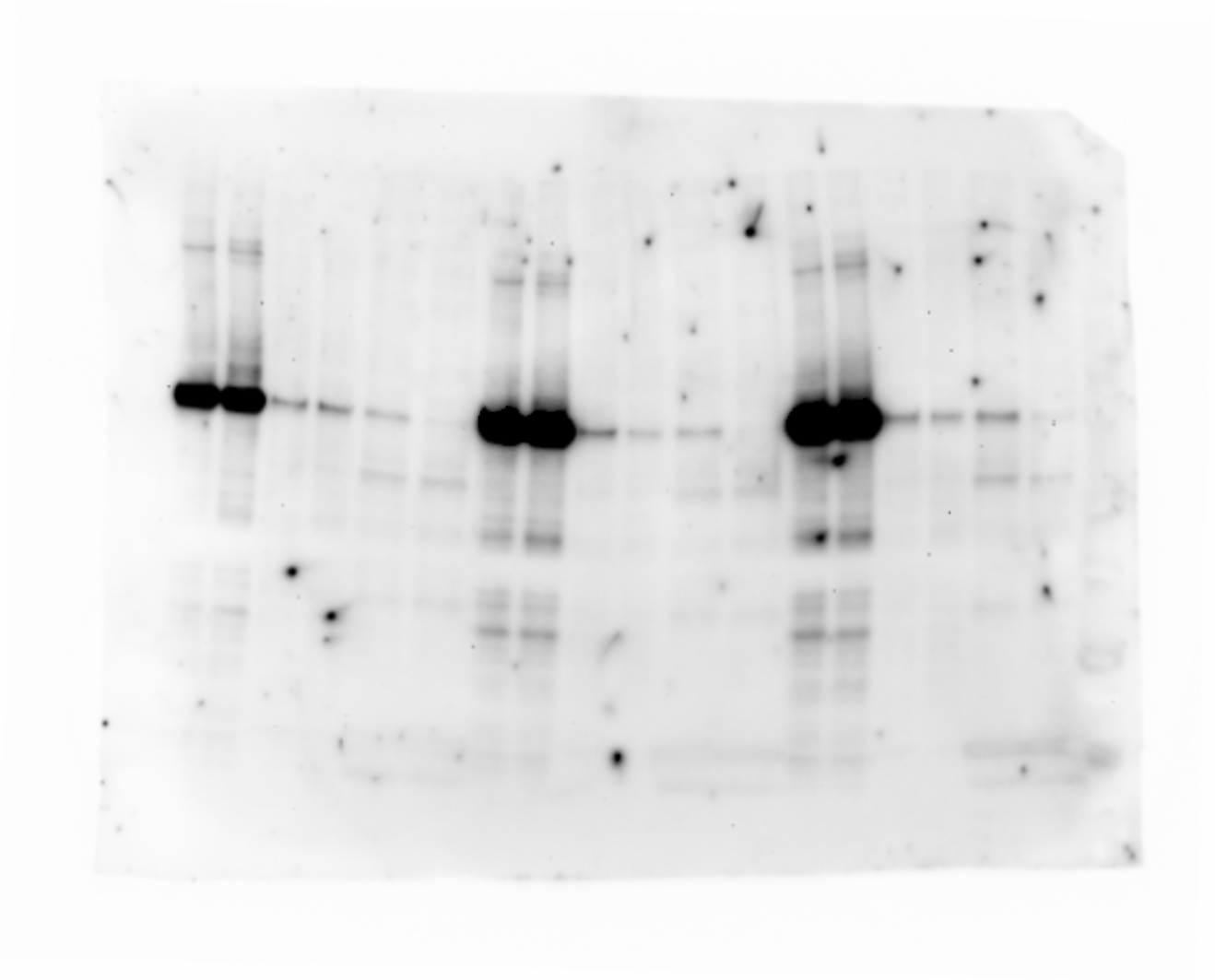

Supplement: Figure 7—source data 1. [file elife-83299-fig7-data1.zip › PFKL_long_10mM-glucose.tif]

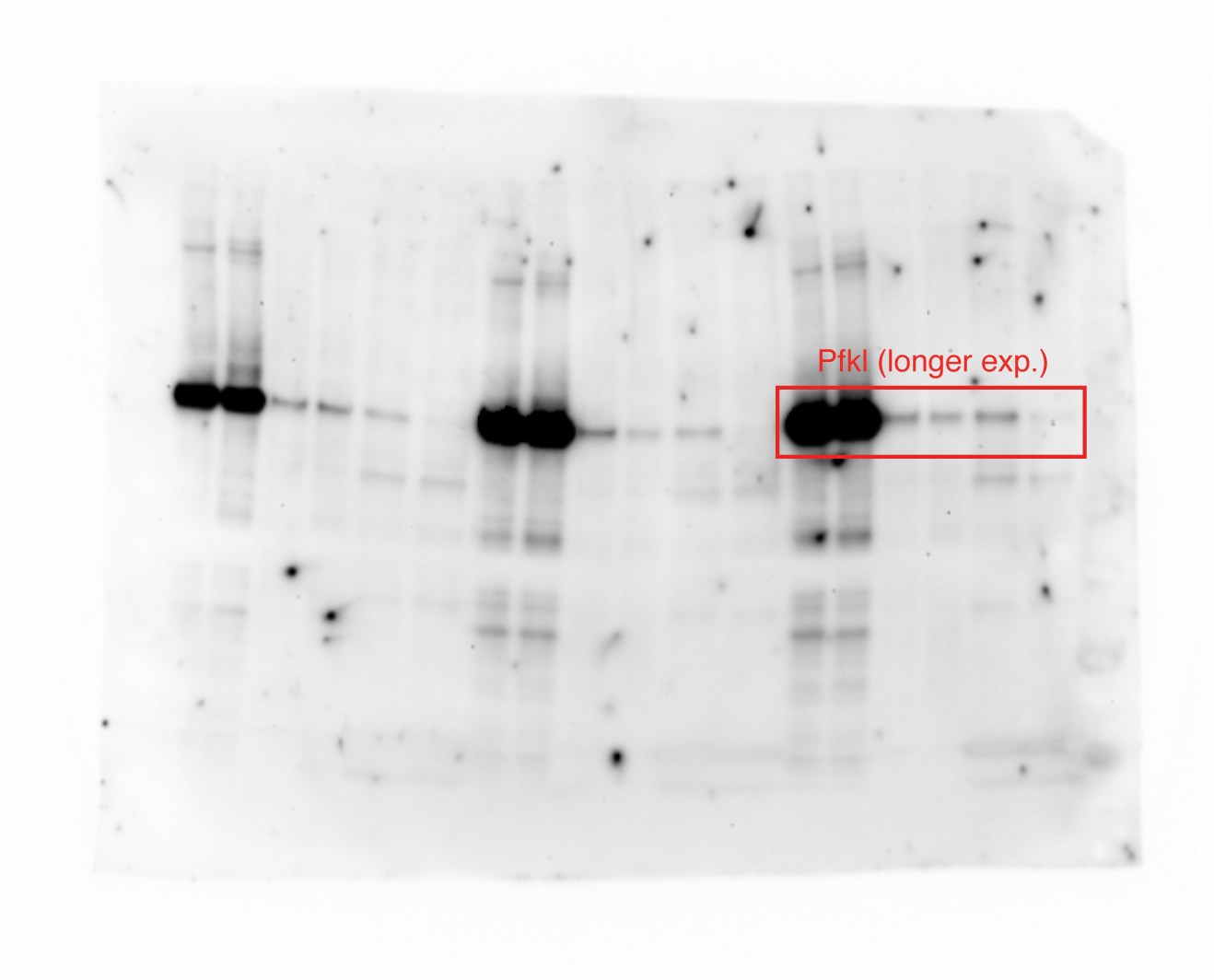

Supplement: Figure 7—source data 1. [file elife-83299-fig7-data1.zip › PFKL_long_10mM-glucose_highlighted.tif]

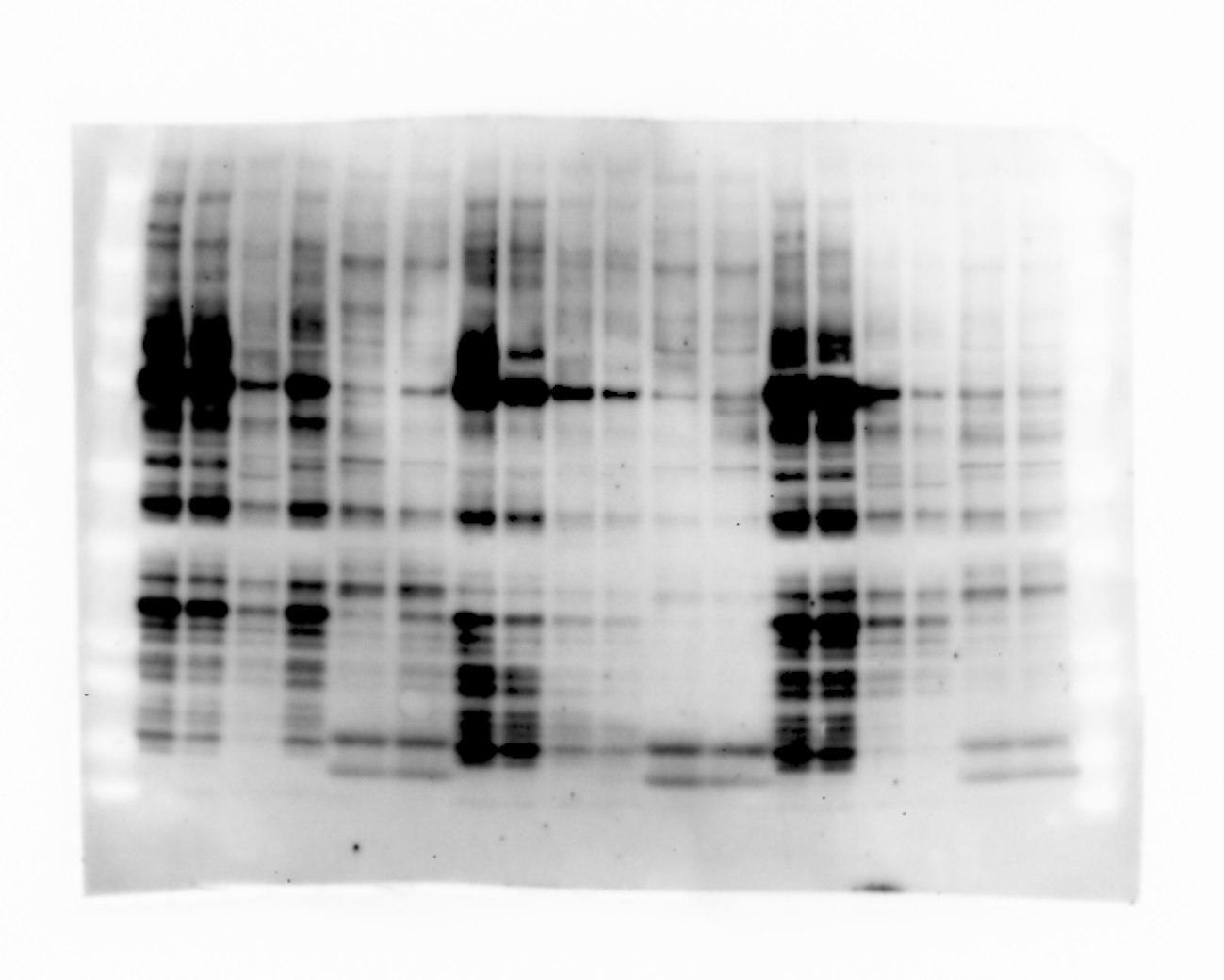

Supplement: Figure 7—source data 1. [file elife-83299-fig7-data1.zip › PFKL_long_2mM-glucose.tif]

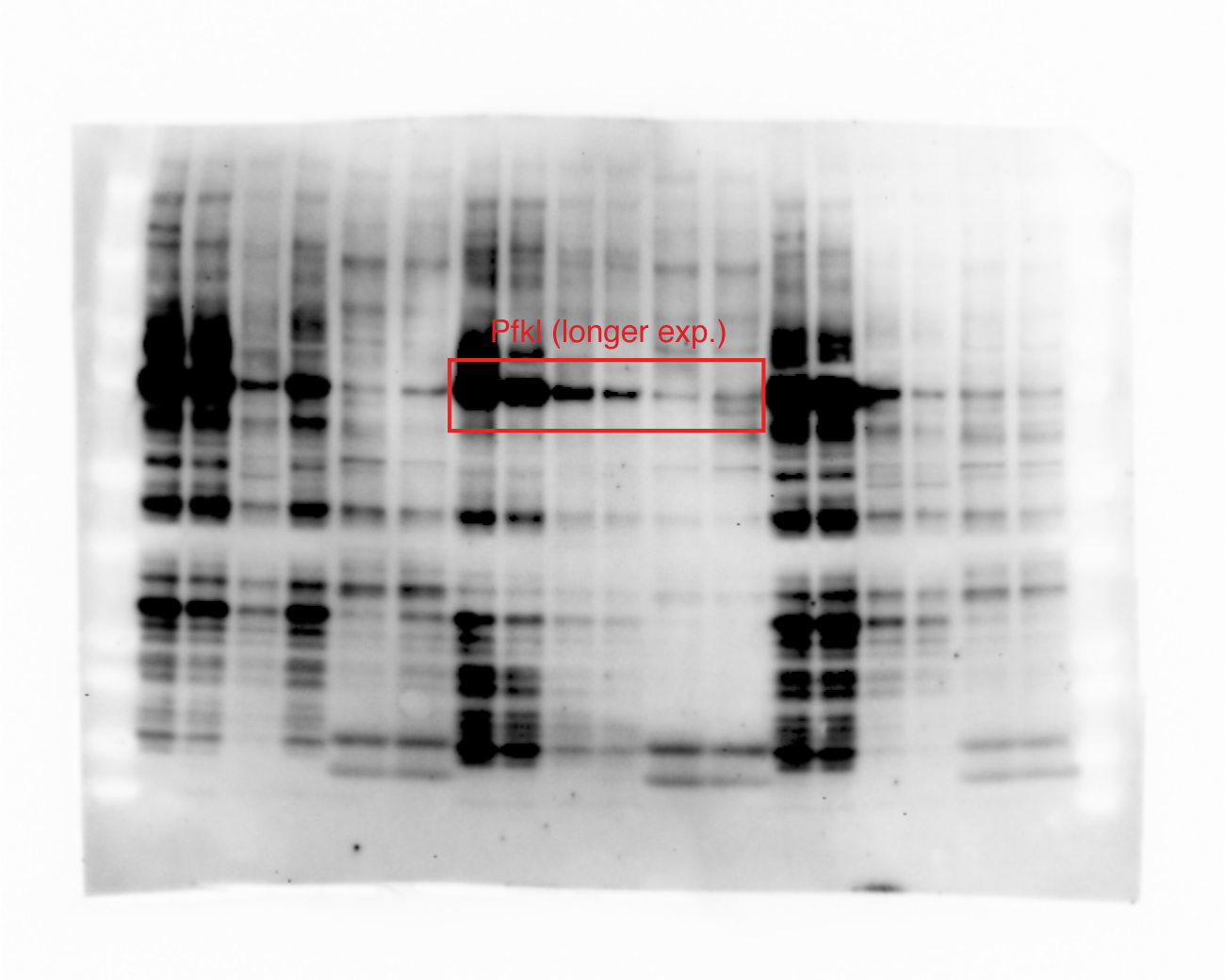

Supplement: Figure 7—source data 1. [file elife-83299-fig7-data1.zip › PFKL_long_2mM-glucose_highlighted.tif]

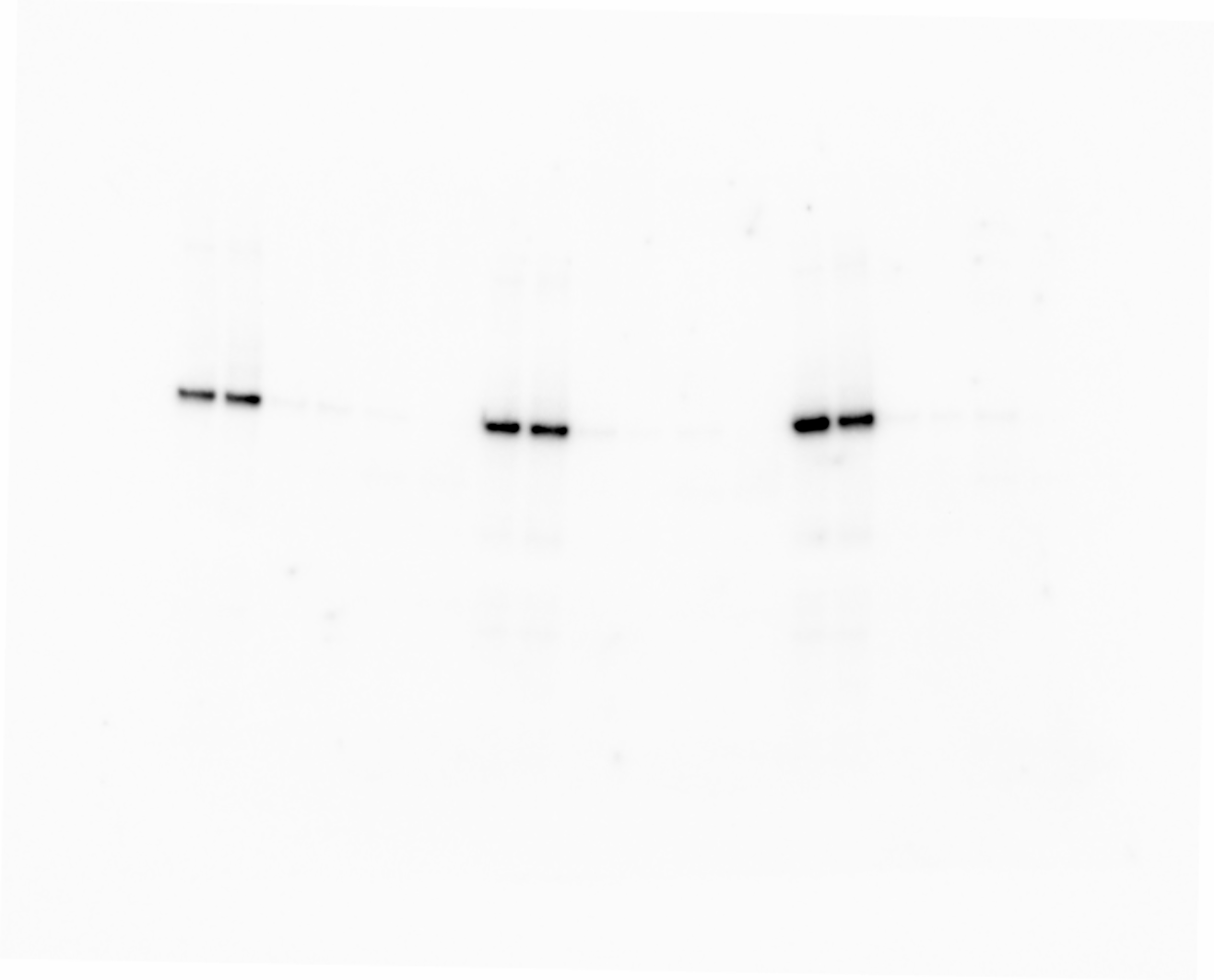

Supplement: Figure 7—source data 1. [file elife-83299-fig7-data1.zip › PFKL_short_10mM-glucose.tif]

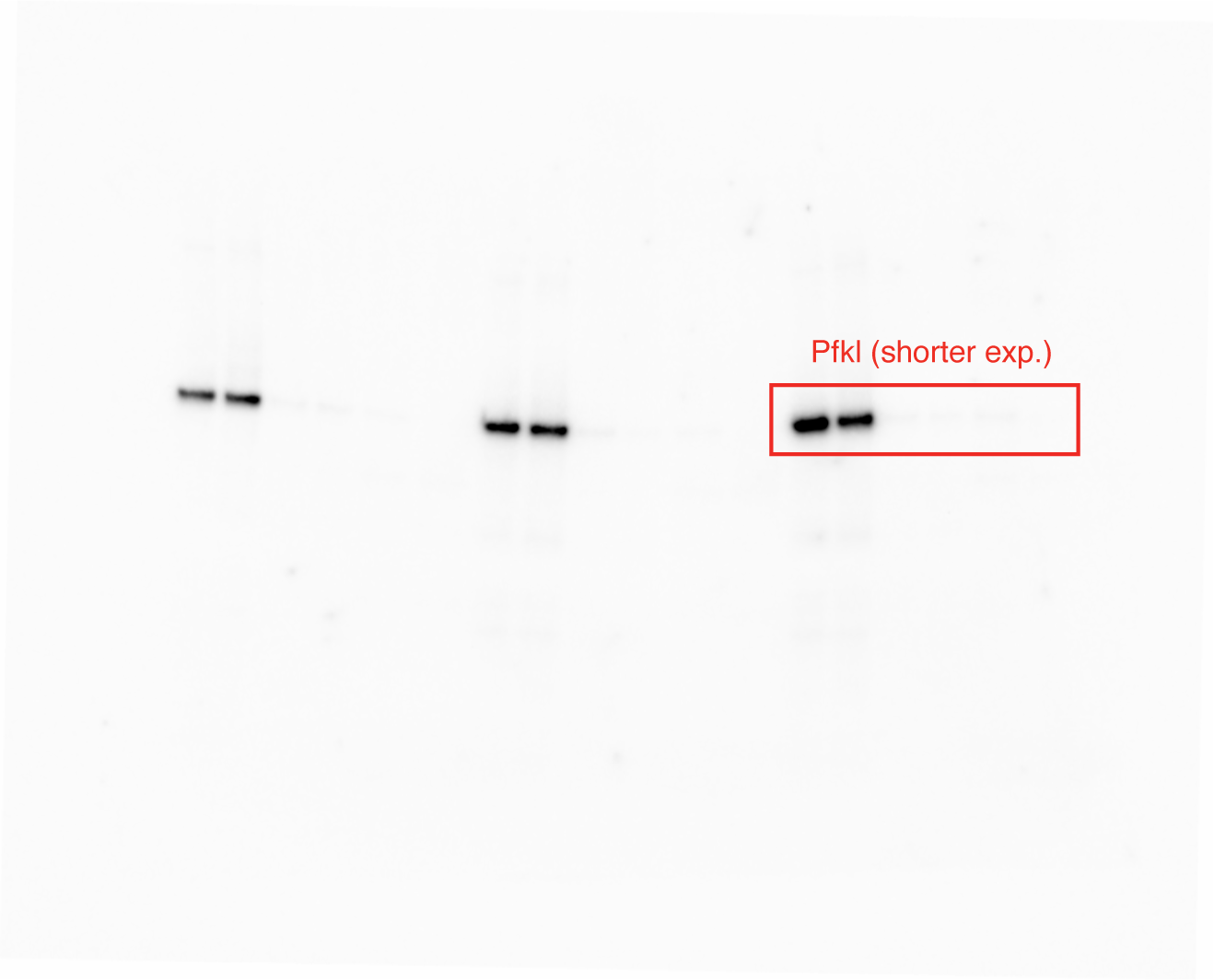

Supplement: Figure 7—source data 1. [file elife-83299-fig7-data1.zip › PFKL_short_10mM-glucose_highlighted.tif]

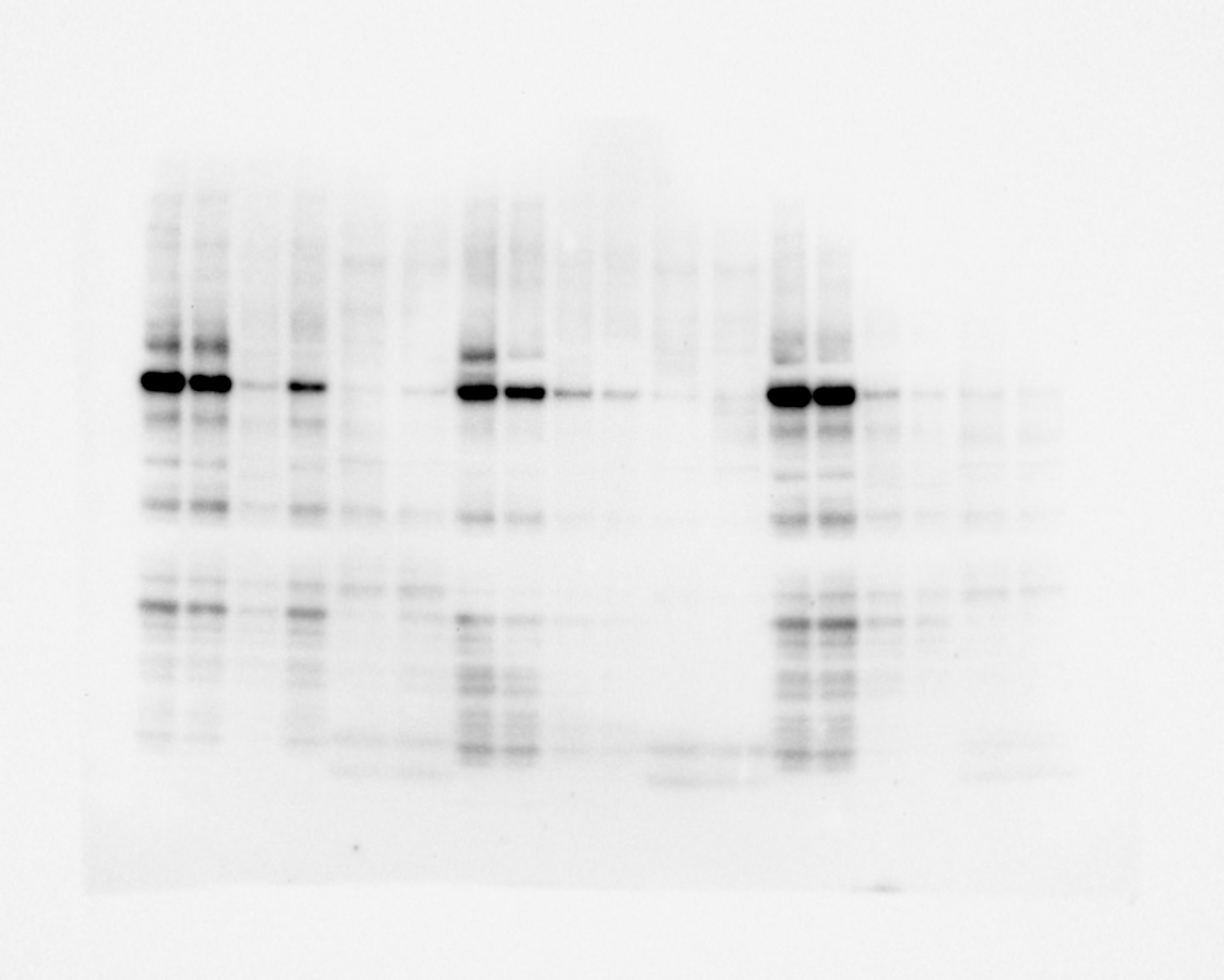

Supplement: Figure 7—source data 1. [file elife-83299-fig7-data1.zip › PFKL_short_2mM-glucose.tif]

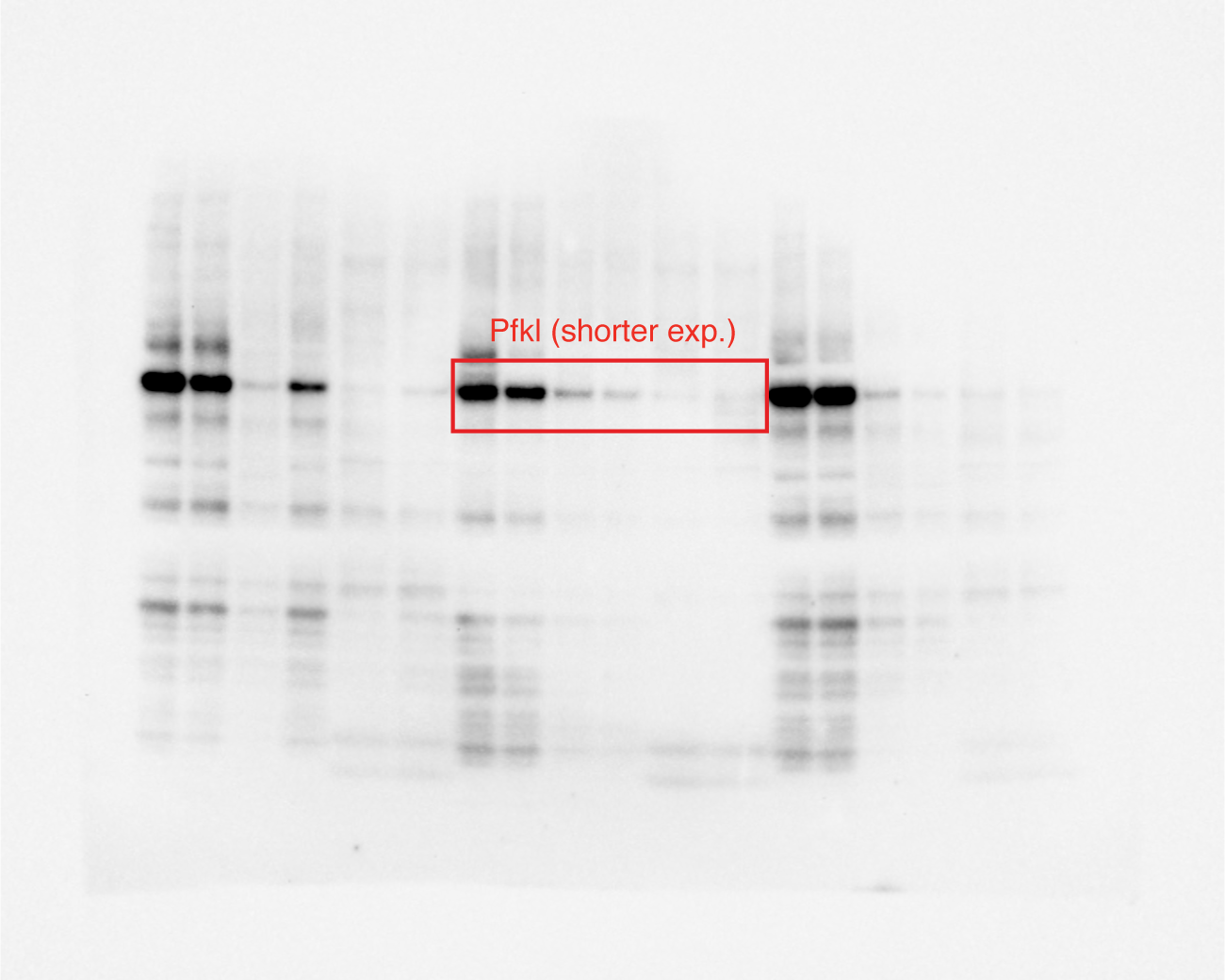

Supplement: Figure 7—source data 1. [file elife-83299-fig7-data1.zip › PFKL_short_2mM-glucose_highlighted.tif]

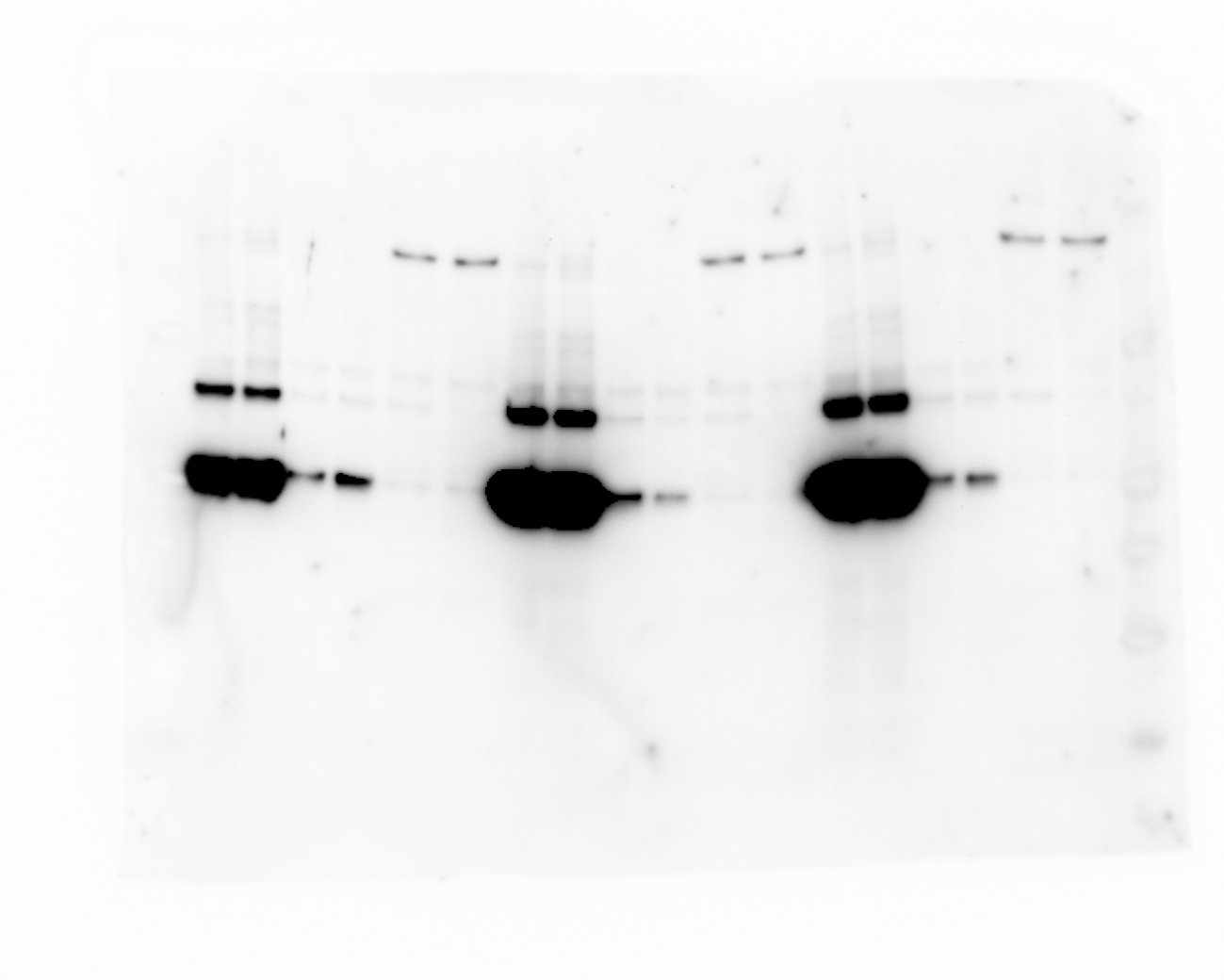

Supplement: Figure 7—source data 1. [file elife-83299-fig7-data1.zip › Top2b_10mM-glucose.tif]

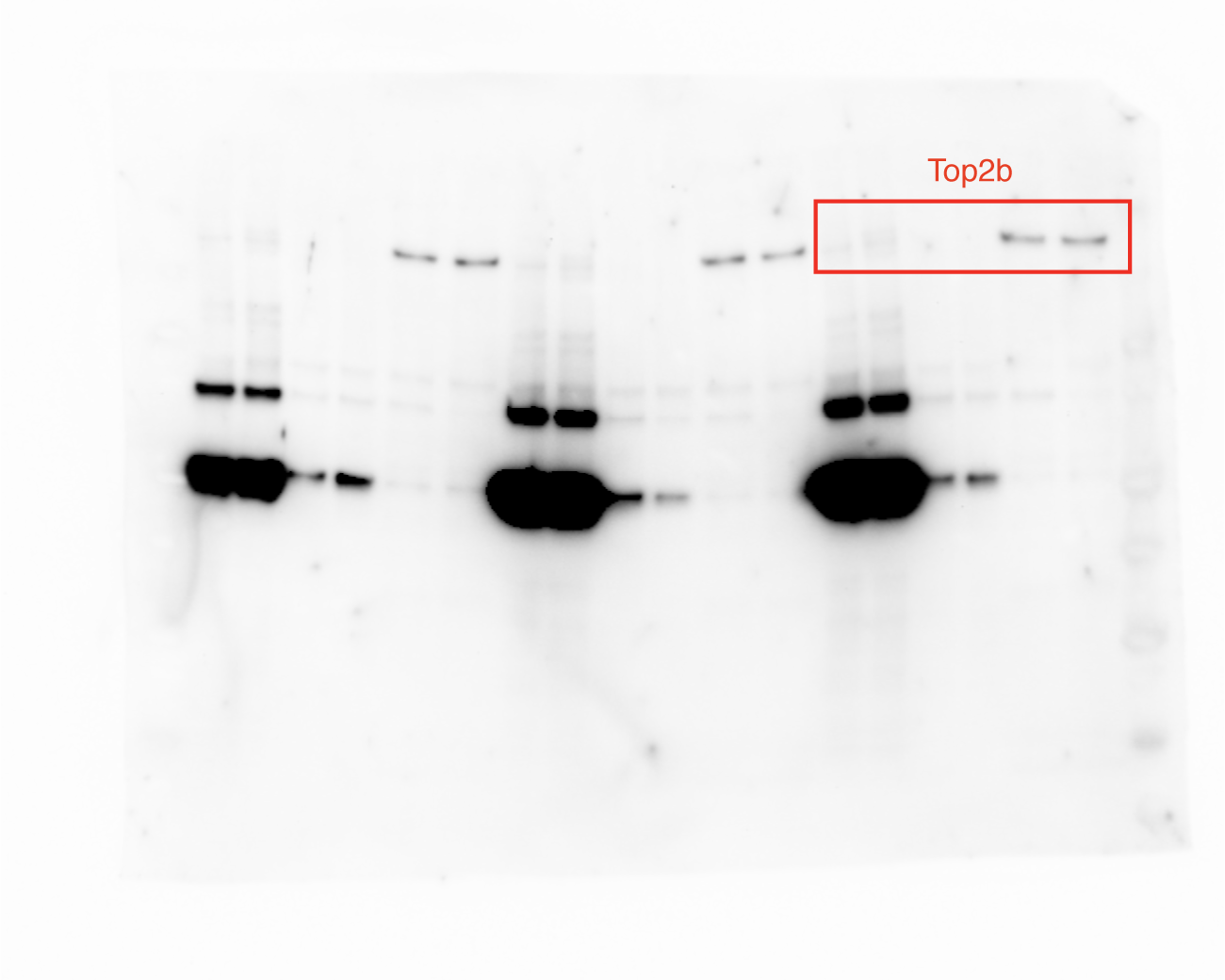

Supplement: Figure 7—source data 1. [file elife-83299-fig7-data1.zip › Top2b_10mM-glucose_highlighted.tif]

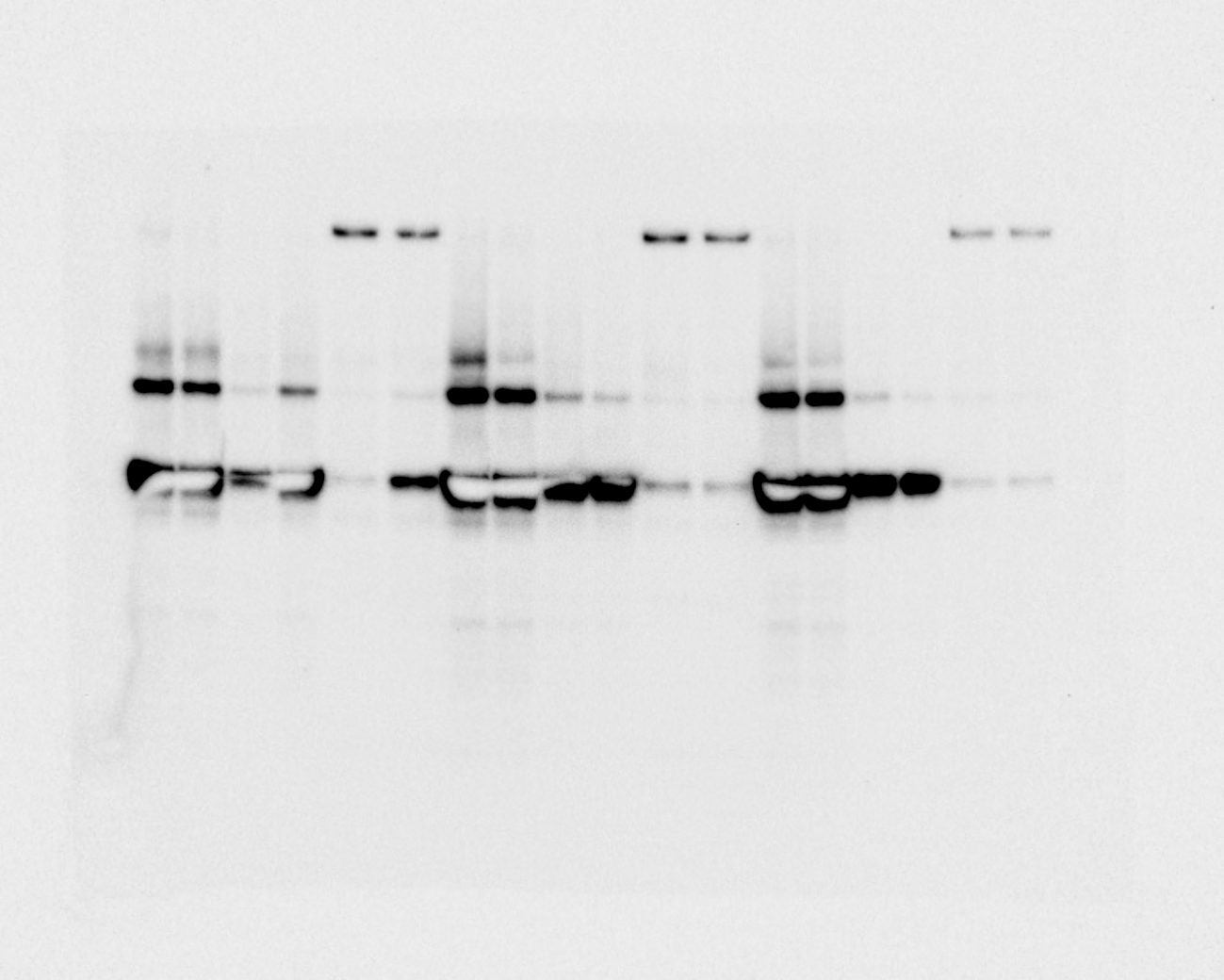

Supplement: Figure 7—source data 1. [file elife-83299-fig7-data1.zip › Top2b_2mM-glucose.tif]

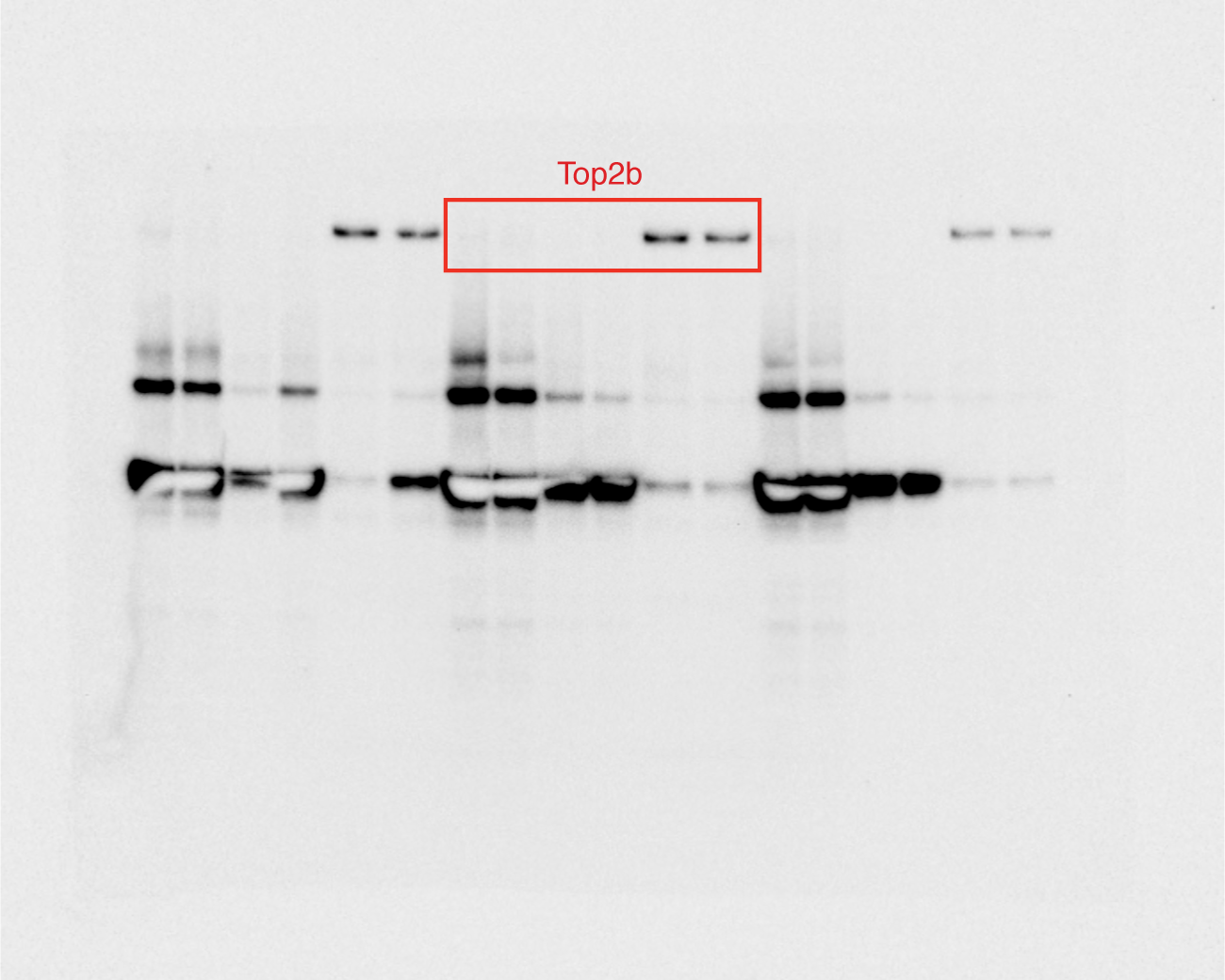

Supplement: Figure 7—source data 1. [file elife-83299-fig7-data1.zip › Top2b_2mM-glucose_highlighted.tif]
